# Metabolic Effects of Selected Conventional and Alternative Sweeteners: A Narrative Review

**DOI:** 10.3390/nu16050622

**Published:** 2024-02-23

**Authors:** Fabienne Teysseire, Valentine Bordier, Christoph Beglinger, Bettina K. Wölnerhanssen, Anne Christin Meyer-Gerspach

**Affiliations:** 1 St. Clara Research Ltd. at St. Claraspital, 4002 Basel, Switzerland; fabienne.teysseire@unibas.ch (F.T.); valentine.bordier@unibas.ch (V.B.); bettina.woelnerhanssen@unibas.ch (B.K.W.); 2 Faculty of Medicine, University of Basel, 4001 Basel, Switzerland; christoph.beglinger@unibas.ch

**Keywords:** glucose, fructose, sucrose, erythritol, xylitol, sucralose, D-allulose, sweeteners, satiation mechanisms, metabolic effects

## Abstract

Sugar consumption is known to be associated with a whole range of adverse health effects, including overweight status and type II diabetes mellitus. In 2015, the World Health Organization issued a guideline recommending the reduction of sugar intake. In this context, alternative sweeteners have gained interest as sugar substitutes to achieve this goal without loss of the sweet taste. This review aims to provide an overview of the scientific literature and establish a reference tool for selected conventional sweeteners (sucrose, glucose, and fructose) and alternative sweeteners (sucralose, xylitol, erythritol, and D-allulose), specifically focusing on their important metabolic effects. The results show that alternative sweeteners constitute a diverse group, and each substance exhibits one or more metabolic effects. Therefore, no sweetener can be considered to be inert. Additionally, xylitol, erythritol, and D-allulose seem promising as alternative sweeteners due to favorable metabolic outcomes. These alternative sweeteners replicate the benefits of sugars (e.g., sweetness and gastrointestinal hormone release) while circumventing the detrimental effects of these substances on human health.

## 1. Introduction

Sugar consumption, particularly that of sucrose (commonly known as table sugar) and glucose–fructose syrups predominantly present in sugar-sweetened beverages (SSBs), has demonstrated a significant increase among both children and adults in recent decades [1,2]. Across Europe, SSBs rank as the second most substantial source of added sugar, following sweet products such as confectionery and sweets [3,4]. In 2015, the World Health Organization (WHO) issued a guideline, both for adults and children, recommending the reduction of sugar intake to less than 10% of total energy intake (TEI) and preferably less than 5% of TEI [5].

**Figure 1:**
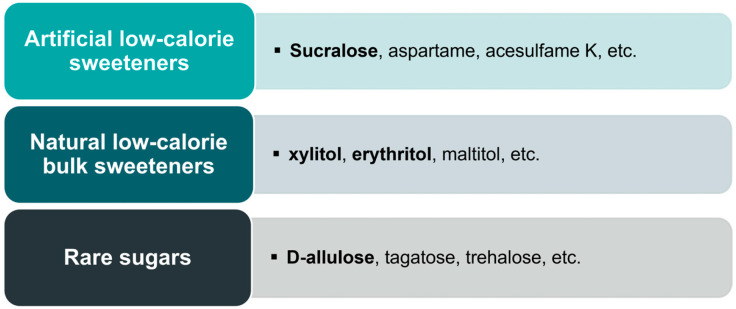
Categories of alternative sweeteners discussed in this review.

In this context, alternative sweeteners have gained interest as substitutes for conventional sweeteners, such as sucrose, glucose, or fructose, to achieve a reduction in sugar intake without loss of the sweet taste. Alternative sweeteners can be categorized into three groups and are discussed in this review: artificial low-calorie sweeteners (LCS), natural low-calorie bulk sweeteners, and rare sugars (see Figure 1).

Just recently, the WHO has released a new guideline on the use of LCS (a category which includes acesulfame K, aspartame, advantame, cyclamate, neotame, saccharin, sucralose, stevia, and stevia derivatives), and cautions against their use as a direct substitution for sugar [6]. Noteworthily, natural low-calorie bulk sweeteners such as erythritol or xylitol, as well as rare sugars such as D-allulose, are not discussed in this new guideline.

Several human studies have shed light on the differential effects of conventional sweeteners (glucose, fructose, and sucrose) and alternative sweeteners on metabolic parameters such as gastrointestinal (GI) hormone secretion, gastric emptying rates, energy intake, glycemic control, blood lipids, and uric acid. These investigations offer valuable insights into their unique metabolic effects.

Previous comprehensive and narrative reviews focused on specific outcomes (e.g, glycemic control, weight, or energy intake), were only covering specific sweetener groups, and/or did not include recent publications—especially those on erythritol, xylitol, or D-allulose [7,8,9,10,11,12]. The aim of this review is to give an overview of the recent scientific literature and build a reference tool for selected conventional and alternative sweeteners from each of the three categories regarding some of their important metabolic and health effects.

## 2. Methods

Relevant manuscripts have been searched thoroughly online in PubMed via Medline, using keywords such as “sucrose”, “glucose”, “fructose”, “xylitol”, “erythritol”, “sucralose”, “d-allulose”, and “sweeteners”, in combination with “metabolic”, “effects”, “hormones”, “gastric emptying”, “satiation”, “energy intake”, “glycemic control”, “blood glucose”, “insulin”, “blood lipids”, and “uric acid”, both as single words and in their various combinations. Only clinical trials with non-diabetic adults were considered. Studies with in vitro, ex vivo, and animal models were not included in this review. Studies for which only the abstract was available, as well as publications not written in English, were also excluded. For each parameter, a table has been created to summarize the effects of the selected LCS on the parameter of interest (see Supplementary Materials). In certain studies, no fixed amounts of sweeteners were administered, but these values were calculated individually according to the body weight or the energy intake of the participants. To unify the results and enable comparisons between studies, the mean body weight, mean energy intake, or, if not available, a standardized body weight of 70 kg was used to calculate the amount of sweeteners administered.

## 3. Effects on Gastrointestinal Hormones

### 3.1. Sucrose, Glucose, and Fructose

Sucrose has been shown to induce the secretion of GI hormones in several acute human studies. Ma et al. [13] reported that intragastric administration of 50 g of sucrose in 500 mL saline led to increased plasma concentrations of glucagon-like peptide-1 (GLP-1) and gastric inhibitory polypeptide (GIP), compared to sucralose or saline solutions in healthy normal-weight participants. Additionally, Yunker et al. [14] investigated the effects of oral consumption of 75 g sucrose in 300 mL water versus sucralose or water, finding that sucrose resulted in an increase in plasma GLP-1, but not peptide tyrosine tyrosine (PYY), and a decreased in ghrelin, compared to sucralose or water, in participants with a body mass index (BMI) of 19.2–40.3 kg/m^2^ [14]. Tai et al. [15] reported that, when compared to water, oral consumption of 100 g of sucrose in 300 mL water led to a reduction in plasma ghrelin concentrations in healthy normal-weight participants. Furthermore, Maersk et al. [16] compared the acute intake of a sucrose beverage (500 mL of regular cola containing 53 g sucrose) to isocaloric milk, an aspartame-sweetened diet cola, and water in participants with overweight status and obesity, observing a reduction in ghrelin and an increase in GLP-1 and GIP concentrations after sucrose.

Steinert et al. [17] examined the effects of intragastric administration of 50 g glucose and 25 g fructose, compared to 62 mg sucralose, 169 mg aspartame, 220 mg acesulfame K (dissolved in 250 mL water), and water alone in healthy, normal-weight individuals. The authors found an increase in GLP-1 and PYY concentrations after glucose, compared to all other substances. Similar results were observed for ghrelin concentrations, with a decrease in ghrelin after glucose but no change after administration of the other substances [17]. Meyer-Gerspach et al. [18], on the other hand, observed an increase in GLP-1 after intragastric administration of both glucose (50 g in 250 mL water) and fructose (25 g in 250 mL water), compared to acesulfame K (220 mg in 250 mL water), and water (only for the glucose). There was no difference in GLP-1 release between glucose and fructose. Moreover, they found that both glucose and fructose induced a significant secretion of cholecystokinin (CCK), as compared to acesulfame K and water. There was no difference in CCK release between glucose and fructose. For ghrelin, their findings show a stronger decrease after glucose compared to that for fructose, with no change after acesulfame K or water.

In addition, other acute studies compared the conventional sweeteners sucrose, glucose, and fructose against each other. Wölnerhanssen et al. [19] observed that an intragastric administration of 75 g of glucose or 25 g of fructose in 300 mL water resulted in an increase in GLP-1 and GIP concentrations, but significantly higher GLP-1 and GIP concentrations after 75 g glucose compared to 25 g fructose. Also, Kong et al.’s study revealed that oral fructose (75 g in 300 mL water) stimulated GLP-1 less than did 75 g glucose [20]. Yau et al. [21] found that sucrose (36 g), glucose (39.6 g), fructose (36 g), and a combination of glucose (19.8 g) with fructose (18 g) (all in 600 mL water) similarly stimulated GLP-1 release in healthy participants (mean BMI 25.5 ± 3.8 kg/m^2^). GIP secretion was induced after sucrose, glucose, and a combination of glucose and fructose, but not after fructose alone. Additionally, lower GIP concentrations were found after sucrose compared to the combination of glucose and fructose. The highest GIP concentrations were found after glucose. Each of the four substances lead to a similar decrease in ghrelin [21]. Furthermore, Yunker et al. [22] compared the effects of 75 g sucrose and 75 g glucose in 300 mL water in healthy participants (mean BMI 27.0 ± 5.0 kg/m^2^), finding that sucrose led to smaller increases in GLP-1 and PYY, compared to glucose, while the decrease in acyl-ghrelin concentrations did not differ between sucrose and glucose [22]. Finally, Matikainen et al. [23] found no change in fasting GLP-1, GIP, and PYY and during an OGTT (GLP-1 and GIP only) before and after a 12-week administration of oral fructose (75 g in 330 mL, 3×/day) in participants with overweight status and obesity. Moreover, there were no changes in GLP-1, GIP, or PYY during a mixed-meal test.

Collectively, these findings suggest that acute intake of sucrose, glucose, and fructose can stimulate the secretion of GI hormones and suppress the secretion of the hunger hormone ghrelin. Chronic intake of fructose appears to have no effect on the secretion of gastrointestinal hormones in response to an OGTT.

### 3.2. Sucralose

Several studies have consistently demonstrated that varying doses of acute sucralose administration (oral, intragastric, or intraduodenal) have had no effect on GI hormones, such as GLP-1, PYY, GIP, or ghrelin [13,14,17,24,25,26,27,28]. However, some studies have reported higher concentrations of GLP-1 during an oral glucose tolerance test (OGTT) when sucralose was given as a preload compared to water [29,30].

A recent study showed that a daily sucralose intake (2 sachets containing 102 mg + 5.898 g glucose as bulking agent, 3×/day) over a two-week period did not lead to changes in GLP-1 concentrations during an OGTT in participants with a BMI between 18–28 kg/m^2^ [31]. In contrast, Lertrit et al. [32] observed an increase in GLP-1 concentrations during an OGTT after consumption of 200 mg/d of sucralose for 4 weeks in healthy participants (BMI: 18.5–27.0 kg/m^2^).

Acute intake of sucralose in isolation has no effect on GI hormone release. When it is given as a preload before an OGTT or when it is given chronically, some studies find an increase in GLP-1 release.

### 3.3. Xylitol and Erythritol

Our recent research has repeatedly demonstrated that acute intragastric or oral administration of varying doses of xylitol (7 to 50 g in 300 mL water) leads to an increase in GLP-1, CCK, and PYY secretion, with no effect on GIP [33,34,35].

Similar results have been observed for erythritol. Acute intragastric or oral administration of varying doses (10 to 75 g in 300 mL water) of erythritol induced the secretion of GLP-1, CCK, and PYY, but not GIP [34,35,36,37,38]. Teysseire et al. [39] found a decrease in plasma ghrelin concentrations in response to an intragastric administration of 50 g of erythritol in 300 mL water, compared to D-allulose and water, in healthy, normal-weight individuals. Sorrentino et al. [40] observed a similar effect following oral consumption of 50.8 g of erythritol in 250 mL water, compared to aspartame in a similar participant population.

In summary, both xylitol and erythritol similarly stimulate the secretion of GLP-1, CCK, and PYY, and have no effect on GIP, irrespective of the dose and administration route. Additionally, erythritol reduces ghrelin secretion. Currently, there is a lack of human studies investigating the effects of xylitol consumption on ghrelin concentrations.

### 3.4. D-Allulose

To date, and to the best of our knowledge, there has been only one relevant human trial, and it studied the acute effects of 25 g D-allulose in 300 mL water on GI hormones, and found an increase in GLP-1, PYY, and CCK, compared to water, with no effect on ghrelin concentrations found in healthy, normal-weight participants [38,39].

## 4. Effects on Gastric Emptying Rates

### 4.1. Sucrose, Glucose, and Fructose

Several acute studies found that sucrose leads to a reduction in gastric emptying rates. For example, Ma et al. [13] demonstrated that intragastric infusions of 50 g of sucrose dissolved in 500 mL saline emptied at a slower rate from the stomach, compared to saline alone and to sucralose solutions, in healthy, normal-weight participants. Similarly, Tai et al. [15] found that gastric emptying rates were reduced in response to an oral sucrose drink (100 g sucrose in 300 mL water), compared to water. Lavin et al. [41] compared the acute administration of a lemon-flavored solution of sucrose (125 g sucrose + 50 mL lemon juice in 450 mL water) to plain water with lemon juice (525 mL water + 50 mL lemon juice) and found that sucrose reduced gastric emptying rates compared to water [41].

Furthermore, Brener et al. [42] found that different concentrations of glucose (0.05, 0.125, 0.25 g/mL, corresponding to 20 g, 50 g, and 100 g of glucose in 400 mL water) emptied at a slower rate compared to saline in healthy participants. In another study performed by Guss et al. [43] in healthy, normal-weight participants, a 10% glucose solution (50 g in 500 mL water) emptied the stomach at a slower rate, compared to water, while the 10% fructose solution (50 g in 500 mL water) had an intermediate effect.

Shi et al. [44] evaluated the effect of oral administration of four solutions with varying concentrations of glucose and sucrose (2, 4, 6, and 8%, corresponding to 9.8, 19.6, 29.4, and 39.4 g of sucrose or glucose, respectively, in 490 mL water) on gastric emptying rates. The authors discovered that all solutions containing either sugar led to a retardation in gastric emptying, compared to water. The glucose solutions exerted a stronger inhibitory effect on gastric emptying compared to the sucrose solutions [44]. In a study carried out by Horowitz et al. [45], participants who received a daily supplementation of 440 g of glucose for 4–7 days experienced an acceleration in gastric emptying rates for both glucose and fructose solution, compared to participants who did not receive daily supplementation. In contrast, 3 days of supplementation with fructose (120 g/d) resulted in an accelerated gastric emptying rate for a fructose, but not a glucose solution, as reported by Yau et al. [46,47].

In summary, acute administration of sucrose, glucose, or fructose prolongs gastric emptying rates, compared to water or saline, with glucose having the most pronounced effect. When given over several weeks, both glucose and fructose can lead to an accelerated gastric emptying rate for acutely administered glucose and fructose solutions.

### 4.2. Sucralose

Sylvetsky et al. [24] found that oral solutions containing different doses of sucralose (68 to 250 g in 355 mL water) had no effect on gastric emptying rates during a subsequent OGTT, compared to water. Also, in another study conducted by Ma et al. [13], intragastric administration of 80 mg or 800 mg of sucralose (dissolved in 500 mL saline) did not result in a significant delay in gastric emptying rates, compared to 50 g of sucrose in 500 mL saline. Furthermore, Wu et al. [26] observed that when different oral preloads (60 mg of sucralose, 40 g of glucose, 40 g of 3-O-Methyl-D-glucose (3-OMG), or 40 g of a tagatose/isomalt mixture; dissolved in 400 mL water), were administered prior to a mashed-potato meal, the gastric emptying rates were slower following the consumption of preloads containing 3-OMG and the tagatose/isomalt mixture, in comparison to sucralose [26].

In short, sucralose has no effect on gastric emptying rates.

### 4.3. Xylitol and Erythritol

Acute intragastric administration of varying doses of xylitol (7 to 50 g in 300 mL water) led to retardation in gastric emptying rates, compared to water, in healthy, normal-weight individuals and participants with obesity [33,35]. In addition, Shafer et al. [48] found that the gastric emptying rates of healthy volunteers were increased after the intake of a radio-labelled meal (scrambled eggs), if the meal was accompanied by a beverage containing 25 g of xylitol (in 50 mL water), compared to a beverage with 25 g of glucose or to water only.

Several studies conducted by our research group (healthy, normal-weight individuals, and participants with obesity) found that intragastric administration of erythritol (at various doses: 10 to 75 g in 300 mL water), compared to water, slows down gastric emptying rates [35,36,38].

To summarize, both xylitol and erythritol lead to prolonged gastric emptying rates compared to water, glucose, or D-allulose.

### 4.4. D-Allulose

To date, and to the best of our knowledge, only one study has assessed the effect of D-allulose on gastric emptying rates. Intragastric administration of 25 g D-allulose dissolved in 300 mL water had no effect on gastric emptying rates in healthy, normal-weight individuals [38].

## 5. Effects on Energy Intake

### 5.1. Sucrose, Glucose, and Fructose

One acute study, from Farhat et al. [49], assessed the effect of a preload containing 60 g of sucrose in 300 mL water, compared to water only, on subsequent energy intake and found no differences, but missed reporting the total energy intake, which would have been higher after the caloric sugar sucrose. Notably, previous research carried out by Rolls et al. [50] indicated that the inclusion of a sucrose drink (20 or 40 g sucrose in lemonade) in a meal leads to a higher total energy intake, compared to water or aspartame, as there were no differences in meal size between the groups. In a study from Melanson et al. [51], the consumption of sucrose and HFCS (both 30% of daily energy intake, corresponding to ca. 134 g of sucrose or 192 g of HCFS) resulted in comparable total daily energy intake levels. Tey et al. [52] reported a reduced subsequent energy intake after 65 g sucrose in 500 mL water, compared to other preloads containing 440 mg aspartame, 0.63 g monk fruit, or 0.33 g stevia, with no differences observed in total energy intake. In contrast to the aforementioned studies, Crézé et al. [53] and Yunker et al. [14] observed a lower total energy intake after a sucrose preload, compared to LCS and/or water, respectively.

Guss et al. [43] looked at subsequent energy intake in response to different (1% and 10%) glucose and fructose solutions flavored with lemon juice, as well as aspartame corresponding to 5 and 50 g glucose and fructose in 500 mL water), compared to water, which were served as a preloads before a test meal. The overall conclusion of this study, conducted with healthy, normal-weight participants, is that the 10% fructose (50 g) and 1% glucose (5 g) solutions reduced total energy intake, compared to water.

Maersk et al. [54] observed no differences in total energy intake before or during a 6-month intervention of daily consumption of sucrose (106 g in 1000 mL water), semi-skimmed milk (47 g carbohydrates in 1000 mL semi-skimmed milk), aspartame-sweetened cola (1000 mL), or 1000 mL water in participants with overweight status or obesity. Kuzma et al. [55], on the other hand, concluded from their two studies that the total energy intake was increased in response to glucose and fructose (25% of daily energy intake for 8 days), compared to aspartame, with no differences found in total energy intake between glucose, fructose, or HFCS. Furthermore, Sigala et al. [56] compared the effect of a chronic intake of glucose, fructose (25% of daily energy intake), HFCS, or aspartame (3×/day for 2 weeks) on energy intake. They found increases in total energy intake in response to glucose, fructose, sucrose, and HFCS, compared to aspartame. Geidl et al. [57] compared the effects of chronic intakes of 79.8 g of either sucrose, glucose, or fructose (3× daily 26.6 g of the conventional sweeteners in 200 mL water for 7 weeks) and found no change in total energy intake. Finally, Reid et al. [58] observed no difference in total energy intake during a 4-week trial in which healthy participants were given beverages containing either sucrose (4×/day 21 g of sucrose in 250 mL water) or aspartame (4×/day 1.78 g of aspartame in 250 mL water).

Taken together, the results of these studies (acute and chronic) are somewhat difficult to interpret, as they all demonstrate mixed outcomes concerning energy intake in response to any of the conventional sweeteners. This difficulty mainly arises from the failure to clearly distinguish between total energy intake and subsequent energy intake, variations in study designs (such as preload dosage and test meal timing, as well as the duration of the study), and differences in the studies’ populations.

### 5.2. Sucralose

Ford et al. [28] found no difference in subsequent energy intake with a buffet meal two hours after consuming sucralose (4.15 mg in 50 mL water) as a preload, compared to water only. In two comparable crossover and parallel-group studies conducted by Gadah et al. [59], a 41.3 g sucrose preload (beverage with a serving volume of 300 mL) induced a reduced subsequent energy intake, compared to a 0.7 g sucralose preload. However, when taking into account the calories of the preload, the total energy intake was lower after sucralose than after sucrose preloads [59]. Akhavan et al. [60] observed that the subsequent energy intake during an ad libitum test meal was similar following a sucralose preload (130 mg), compared to two different sugar preloads (75 g sucrose, or a mixture of 37.5 g glucose and 37.5 g fructose in 300 mL water) in healthy participants. However, the total energy intake, considering the calories from the sugar preloads, was higher compared to the sucralose preload. In another study by Chern et al. [61], a preload with 120 mg sucralose, compared to either 50 g sucrose or a mixture of 120 mg sucralose and maltodextrin matched in carbohydrate content with 50 g sucrose, resulted in higher energy intake, when total energy intake was recorded for the remainder of the day. Consequently, the lower energy content of the preload was fully compensated for [61]. Yunker et al. [14] reported a higher total energy intake after a certain sucralose preload (178–358 mg, individually matched, and dissolved in 300 mL water) compared to a 75 g sucrose preload. Furthermore, Higgins and Mattes [62] observed a decreased total energy intake over 12 weeks in response to a daily sucralose drink (160 mg) compared to a sucrose drink (100, 120, or 140 g) in adults with overweight status or obesity. The difference in energy intake was attributed to the calories in the sucrose drink, and when these calories were removed, the effect was no longer statistically significant.

To summarize, a clear consensus regarding whether a preload of sucralose increases or decreases subsequent energy intake remains elusive due to some studies having failed to clearly distinguish between total energy intake and subsequent energy intake.

### 5.3. Xylitol and Erythritol

Shafer et al. [48] found that healthy volunteers given a preload of 25 g of xylitol in 50 mL water had a reduced subsequent energy intake, compared to volunteers given a pre-load of 50 mL water only. Additionally, in a recent study from our group (Flad et al. [63], unpublished data), a preload of 33.5 g xylitol in 300 mL water led to reduced total energy intake, compared to a preload of 33.5 g sucrose.

In a study by Teysseire et al. [37], the administration of a pure oral erythritol (50 g in 300 mL water) preload translated into a reduction in subsequent energy intake during an ad libitum test meal, but also into a reduction in total energy intake, compared to other preloads containing sucrose (33.5 g), sucralose (55.8 mg), or water in healthy, normal-weight individuals.

In short, both xylitol and erythritol given as an oral preload elicit a reduction in subsequent energy intake.

### 5.4. D-Allulose

To date, and to the best of our knowledge, no clinical trial on the effect of D-allulose on energy intake exists.

## 6. Effects on Glycemic Control

### 6.1. Sucrose, Glucose, and Fructose

A study carried out by Tai et al. [15] in healthy, normal-weight individuals found that oral sucrose ingestion (100 g in 300 mL water) led to elevated blood glucose and plasma insulin concentrations, in comparison to water. Similar effects were observed by Yunker et al. [14] (75 g sucrose in 300 mL water versus water or sucralose) and Ma et al. [13] (50 g sucrose in 500 mL saline versus saline or sucralose).

In another study performed by Tey et al. [52], acute intake of a sucrose-sweetened beverage (65 g sucrose in 500 mL water, compared to 0.33 g stevia, 440 mg aspartame, or 0.63 g monk-fruit in 500 mL water) resulted in significant increases in blood glucose and insulin concentrations within the time prior to an ad libitum test lunch. As there was a higher subsequent energy intake after the preload containing stevia, aspartame, or monk-fruit, compared to the sucrose beverage, higher blood glucose and insulin responses were found after the test lunch with the three low-calorie sweeteners. Consequently, there were no differences in the overall AUC for glucose and insulin concentrations among the four tested beverages [52].

Furthermore, Meyer-Gerspach et al. [18] showed that the intragastric administration of both 50 g glucose and 25 g fructose in 250 mL water increased plasma glucose concentrations, compared to acesulfame K and water. However, the glucose solution led to a stronger increase in plasma glucose, compared to fructose [18].

Yau et al. [21] found a higher incremental AUC for glucose after administration of oral solutions containing 39.6 g glucose, 36 g sucrose, or a combination of 19.8 g glucose and 18 g fructose, compared to a solution of 36 g fructose in 600 mL water. The AUC for insulin was the highest after the glucose solution, followed by the sucrose solution and the combination of glucose and fructose, whereas the fructose solution led to the smallest insulin AUC. Kawakami et al. [64] found a greater increase in plasma glucose after 50 g sucrose and 50 g isomaltulose, compared to 25 g fructose alone (all drinks were constituted in water to a final volume of 250 mL). Similarly, Jameel et al. [65] reported that the oral intake of 50 g sucrose and 50 g glucose in 300 mL water led to a stronger increase in blood glucose and insulin concentrations, compared to 50 g fructose, in healthy, normal-weight individuals.

Wölnerhanssen et al. [19] showed that an intragastric administration of 75 g of glucose in 300 mL water resulted in a significant increase in blood glucose and insulin concentrations, while 25 g of fructose had no impact. Kong et al. [20], on the other hand, showed that both a 75 g glucose solution and a 75 g fructose solution (in 300 mL water) resulted in increases in blood glucose and insulin. However, this increase was significantly higher after oral ingestion of glucose, compared to fructose. Another study by Eckstein et al. [58] investigated the effects of oral administration of glucose (76.3 g in 300 mL water), fructose (76.3 g in 300 mL water), glucose and fructose (38.2 g, respectively, in 300 mL water), or sucralose (0.2 g in 300 mL water) on blood glucose and insulin concentrations. They found that glucose, glucose and fructose, and fructose each increased blood glucose and insulin, whereas sucralose had no effect. Blood glucose and insulin concentrations were significantly lower in response to fructose, compared to glucose, as well as glucose and fructose, with no difference found between the last two treatments. Also, when comparing 75 g of sucrose to 75 g glucose in 300 mL water, Yunker et al. [22] found that both substances increased blood glucose and insulin concentrations, and that the AUCs for blood glucose and insulin concentrations were higher after glucose, compared to sucrose, in healthy participants with overweight status.

Sigala et al. [66] compared the chronic intake of beverages containing ca. 147 g sucrose in 980 mL water with that of either ca. 150 g HFCS in 1000 mL water (providing 25% of each subject’s daily energy requirement), or ca. 375 mg aspartame in 1059 mL water, daily, for two weeks. The authors found that the sucrose and HFCS beverages led to a decrease in the Matsuda insulin sensitivity index, compared to the aspartame beverage. Additionally, both the sucrose and the HFCS beverages resulted in similar increases in insulin concentrations during an OGTT, whereas aspartame had no impact. Fasting blood glucose and insulin concentrations remained unchanged after chronic intake of all three different beverages. In a study conducted by Stanhope et al. [67], the chronic consumption of glucose and fructose beverages, each providing 25% of daily energy intake, over a 10-week period was investigated in participants with overweight status and obesity. Results indicated a decrease in fasting blood glucose and an increase in fasting blood insulin concentrations in response to the glucose beverages. During the OGTT there were increases in blood glucose, insulin, and the insulin sensitivity index after chronic glucose intake. Conversely, for the fructose beverage, elevated concentrations of blood glucose and insulin were observed both in fasting and during an OGTT and were accompanied by a decreased insulin sensitivity index during the OGTT. Maersk et al. [54] found no changes in fasting blood glucose, insulin and HOMA-IR in response to a chronic intake (6 months) of 53 g sucrose, semi-skimmed milk, or aspartame-sweetened cola, in similar populations. Also, Geidl et al. [57] found no changes in fasting blood glucose, insulin, HOMA-IR, and blood glucose/insulin concentrations, during an OGTT, in healthy participants consuming 79.8 g sucrose, glucose, or fructose (3× daily 26.6 g of the conventional sweeteners, in 200 mL water) over 7 weeks. Lastly, Matikainen et al. [23] observed no differences in fasting blood glucose, insulin, HOMA-IR, or the Matsuda index during an OGTT in participants with overweight status and obesity who consumed oral fructose (25 g in 330 mL, 3×/day; total, 75 g/day) for 12 weeks. In summary, all conventional sweeteners induce an increase in blood glucose concentrations when consumed acutely and in isolation. Glucose seems to have the strongest effect, followed by sucrose, and finally by fructose. Regarding studies in which these substances were given over a longer period of time, the results are more heterogeneous, some indicating that conventional sweeteners seem to impair glycemic control.

### 6.2. Sucralose

The vast majority of acute and chronic studies conducted in healthy participants found no effect on glycemic outcomes (glucose, insulin, C-peptide, or glycated hemoglobin (HbA1c)) in response to the oral, intragastric, or intraduodenal administration of differing dosages of sucralose [13,14,17,24,25,26,27,28,29,68,69,70,71].

Studies examining the effects of sucralose combined with carbohydrate ingestion have yielded mixed results. According to Temizkan et al. [30], ingestion of 24 mg sucralose in 200 mL water before an OGTT led to lower blood glucose concentrations during an OGTT, compared to water, in healthy humans. However, no effect was observed on insulin or C-peptide concentrations. Lertrit et al. [32] observed a decrease in insulin sensitivity (Matsuda index) and an increase in insulin resistance (HOMA-IR) after consumption of capsules containing 200 mg of sucralose over a four-week period, compared to empty capsules. A more recent study by Dalenberg et al. [72] demonstrated that short-term (10 days) consumption of 60 mg sucralose combined with 31.8 g maltodextrin in 355 mL water decreased insulin sensitivity, whereas daily intake of 60 mg sucralose or 30.4 g sucrose in 355 mL water alone did not alter insulin sensitivity. Suez et al. [31] examined the effect of daily supplementation with 612 mg sucralose and 35.4 g glucose over 2 weeks, compared to that of 35.4 g glucose alone, and found a stronger increase in blood glucose concentrations during an OGTT when sucralose was combined with glucose, compared to glucose alone, in healthy participants with a BMI range of 18–28 kg/m^2^.

Taken together, neither acute nor chronic pure sucralose consumption has a significant effect on glucose homeostasis or HbA1c. However, when sucralose is taken in combination with other carbohydrates, which is closer to real-life conditions, the results are heterogenous. Therefore, more research is needed on investigating the effects of sucralose combined with other carbohydrates on glycemic control.

### 6.3. Xylitol and Erythritol

Several acute studies conducted with healthy, normal-weight participants and participants with obesity showed small increases in blood glucose and insulin concentrations after oral or intragastric administration of various doses of xylitol (7 to 50 g of xylitol in 250–300 mL water) [33,34,35,73,74]. However, the observed increase was significantly lower than after equally sweet loads of glucose. Finally, a chronic oral administration of xylitol (24 g/day) for five weeks had no effect on blood glucose or insulin concentrations during an OGTT or on insulin resistance (HOMA-IR), in participants with obesity but who were otherwise healthy [75].

Several acute studies conducted with healthy, normal-weight participants and participants with obesity did not find increased glucose or insulin concentrations in response to various doses (10–75 g in 250–300 mL water, or as solid snack) of erythritol administered either orally or intragastrically [34,35,36,37,39,76,77]. Finally, a chronic administration of erythritol (36 g/day) for five weeks had no effect on blood glucose and insulin concentrations during an OGTT, or on insulin resistance (HOMA-IR) in healthy participants with obesity [75].

In short, acute xylitol administration leads to small rises in glucose and insulin concentrations and chronic intake has no impact on blood glucose and insulin concentrations during an OGTT, or on insulin resistance (HOMA-IR). Erythritol has no effect on glucose and insulin concentrations, regardless of dose, route, and duration of administration.

### 6.4. D-Allulose

A number of studies, both acute and chronic, reported that oral or intragastric administration of different doses of D-allulose, either in isolation or in combination with other carbohydrates, resulted in either no change [78,79,80] or a decrease in blood glucose concentrations [39,81,82]. In addition, when combined with sucrose or maltodextrin, increasing doses of D-allulose had a dose-dependent reductive effect on glucose and insulin concentrations, compared to sucrose or maltodextrin alone [80,82].

## 7. Effects on Blood Lipids

### 7.1. Sucrose, Glucose, and Fructose

A study carried out by Jameel et al. [65] compared the acute administration of 50 g sucrose, 50 g glucose, and 50 g fructose in 300 mL water and revealed that fructose led to significantly higher concentrations of total cholesterol, low-density lipoprotein cholesterol (LDL), and high-density lipoprotein cholesterol (HDL), compared to glucose and sucrose. However, there were no significant differences in triglyceride concentrations between glucose, fructose, and sucrose [65]. Yau et al. [46] performed another acute study and compared oral beverages containing 39.6 g glucose, or 36 g fructose, in 600 mL water with and without prior fructose supplementation (3 days with 120 g/d of fructose), in healthy participants with a mean BMI of 25.5 ± 3.8 kg/m^2^. They found no effects on blood triglyceride concentrations after consumption of either the glucose or the fructose beverages. In contrast, a study from Chong et al. [83] found that the acute ingestion of a 52.5 g fructose, compared to a 52.5 g glucose, test meal (corresponding to 0.75 g of fructose or glucose per kg body weight) in healthy volunteers (BMI 22 to 31 kg/m^2^) resulted in higher post-prandial plasma TG concentrations after fructose, compared to glucose.

In another chronic study from Bantle et al. [84], diets in which 17% of the total energy intake consisted of either fructose (corresponding to ca. 85 g/d) or glucose (corresponding to ca. 81 g/d), consumed over 6 weeks, were compared in healthy participants. The fructose diet resulted in higher fasting, as well as post-prandial and daylong, TG concentrations than the glucose diet in men, but not in women. Additionally, no differences in fasting total cholesterol, LDL or HDL concentrations were noticed in either men or women. Moreover, Lê et al. [85] observed that 4 weeks of a high-fructose diet (1.5 g/kg body weight/d of fructose; corresponding to ca. 103 g/d of fructose) in healthy participants resulted in increased fasting TG concentrations compared to baseline. Sigala et al. [66] found that daily consumption of beverages sweetened with sucrose or HFCS (providing 25% of each subject’s daily energy requirement; corresponding to 147 g sucrose in 980 mL water or 150 g HFCS in 1000 mL water) for two weeks resulted in elevated fasting concentrations of LDL and TG, compared to aspartame [66]. Similar changes in lipid metabolism have been observed in two other chronic studies performed by Stanhope et al. [67] (increase in 23 h AUC for TG after fructose intake compared to glucose) and Maersk et al. [54] (increase in fasting total cholesterol and fasting TG after sucrose compared to milk or aspartame), respectively. Geidl et al. [57] found no changes in fasting total cholesterol, LDL, HDL, or TG in healthy male participants consuming 79.8 g sucrose, glucose, or fructose (3× daily 26.6 g of the conventional sweeteners in 200 mL water) over 7 weeks. However, after sucrose treatment, elevated concentrations of small, dense low-density lipoprotein particles (sdLDLs) were found [57]. Finally, the study by Matikainen et al. [23] showed an increase in fasting TG concentrations as well as an increase of TG concentrations during a mixed-meal test after daily consumption of 25 g of fructose dissolved in 330 mL water (3×/day), over a period of 12 weeks in participants with overweight status and obesity.

In summary, fructose seems to induce the strongest increases in total cholesterol, LDL, HDL, and TG concentrations. Sucrose also has an effect, especially on total cholesterol and LDL, whereas glucose seems to have no effect, or very little effect, on blood lipids.

### 7.2. Sucralose

The chronic administration of sucralose (2 mg/kg body weight/d or 5 mg/kg body weight/d; corresponding to around 140 mg/d and 350 mg/d, respectively, over 17 days in total, including progressive increases in the concentrations) did not result in significant differences in total cholesterol or triglycerides concentrations [70].

### 7.3. Xylitol and Erythritol

Meyer-Gerspach et al. [33] observed no changes in total cholesterol, LDL, or HDL concentrations after intragastric xylitol administration (7, 17, and 35 g in 300 mL water) compared to water only. However, in their study, xylitol had no effect on TG concentrations. Finally, a chronic oral administration of xylitol (24 g/day) for five weeks had no effect on total cholesterol, HDL, or TG concentrations in participants with obesity, but otherwise healthy, while LDL concentrations decreased over time; however, this was also seen in the control group, which ingested no substances [75].

Acute studies did not find any significant changes in total cholesterol, LDL, HDL, or TG concentrations in response to different doses (10 to 50 g in 300 mL water) of erythritol (intragastric administration) in healthy individuals [36,39]. In addition, a chronic oral intake of 36 g/d of erythritol for five weeks resulted in no changes in total cholesterol, HDL, or TG, and decreased LDL concentrations in participants with obesity; however, this was also seen in the control group, which ingested no substances [75].

Considering the data as a whole, xylitol has demonstrated no impact on total cholesterol, LDL, or HDL concentrations, except for one study that reports an increase in TG after acute enteral xylitol administration. Erythritol shows no effect on blood lipid concentrations, in both acute and chronic settings.

### 7.4. D-Allulose

Teysseire et al. [39] found that acute intragastric administration of D-allulose (25 g in 300 mL water), compared to water, had no effect on blood lipids (total cholesterol, LDL, HDL, or TG). In addition, Kimura et al. [81] did not find any changes in total cholesterol and triglycerides during a test meal, after administration of a 5 g D-allulose in 150 mL water preload, compared to a 10 mg aspartame in 150 mL water preload. Furthermore, Hayashi et al. [78] did not observe any changes in total cholesterol, LDL, and TG concentrations after 12 weeks of daily consumption of 5 g of D-allulose or glucose (in 200 mL tea, consumed together with meals 3×/day) in healthy, normal-weight participants.

In short, both acute and chronic administrations of D-allulose have no effect on blood lipid concentrations.

## 8. Effects on Uric Acid

### 8.1. Sucrose, Glucose, and Fructose

Wolyniec et al. [86] found an increase in uric acid after oral administration of sucrose, glucose, fructose, or xylitol (for all, 35 g in 500 mL water) in healthy participants. This increase was the highest after xylitol, and was similar after the three conventional sweeteners. Kawakami et al. [64] investigated the effects of oral administration of 25 g fructose, 50 g sucrose, and 50 g isomaltulose, in ca. 250 mL water, on uric acid concentrations and found a greater increase in response to fructose compared to sucrose or isolmaltulose in healthy participants.

Sigala et al. [66] demonstrated that consuming beverages sweetened with sucrose or HFCS (providing 25% of each subject’s daily energy requirement, corresponding to 147 g sucrose in 980 mL water, and 150 g HFCS in 1000 mL water) on a daily basis for two weeks led to an increase in total 24 h AUC uric acid concentrations, compared to aspartame, in healthy individuals. These observations are in line with a long-term study (6 months) with participants with overweight status and obesity, conducted by Bruun et al. [87], in which the participants drank 1 L of sucrose-sweetened cola (corresponding to 106 g of sucrose) per day. They found that the sucrose-sweetened cola increased fasting uric acid concentrations, whereas other beverages, such as isocaloric semi-skimmed milk (containing 47 g carbohydrates in 1 L), or aspartame-sweetened diet cola (1 L), had no effect on uric acid concentrations [87]. Moreover, Cox et al. [88] investigated the effects of a daily fructose or glucose beverage (also 25% of each subject’s daily energy requirement) for 10 weeks, in participants with overweight status or obesity. The authors found increased fasting uric acid concentrations after 10 weeks, compared to baseline, after both fructose and glucose, but the effect was greater after fructose.

Collectively, fructose appears to exert the most significant influence on uric acid concentrations, followed by sucrose, while glucose has only a minimal impact.

### 8.2. Sucralose

Repeated daily exposure to different dosages (125 mg to 500 mg) of sucralose for up to 12 weeks had no effect on uric acid concentrations in healthy individuals [70].

### 8.3. Xylitol and Erythritol

It has been shown that acute ingestion of 35 g of xylitol in 300 mL or 500 mL water leads to an increase in uric acid concentrations in healthy volunteers [33,86]. However, a chronic administration of xylitol (24 g/day) for five weeks had no effect on uric acid concentrations in participants with obesity, but who were otherwise healthy [75].

The acute or chronic consumption of erythritol (at various doses from 10 to 50 g in 300 mL water; 36 g/d for five weeks) did not induce increases in uric acid concentrations in healthy, normal-weight participants [36,39], or in participants with obesity [75].

The acute intake of xylitol leads to an elevation in uric acid concentrations; however, chronic intake shows no effect. Erythritol has no impact on uric acid concentrations.

### 8.4. D-Allulose

Teysseire et al. [39] found no changes in uric acid concentrations in response to an acute administration of 25 g of D-allulose in 300 mL water, compared to water, in healthy, normal-weight individuals.

In a 12-week trial with daily D-allulose vs. glucose consumption (3 times per day, 5 g of d-allulose or glucose in 200 mL tea, together with meals), Hayashi et al. [78] found no impacts on blood concentrations of uric acid after either substance.

In summary, acute or chronic D-allulose administration at different doses up to 25 g does not lead to an increase in uric acid concentrations.

## 9. Discussion

The World Health Organization (WHO) has recommended that sugar consumption should be reduced [5]. To achieve this goal without having to completely give up the sweet taste, conventional sweeteners (glucose, fructose, and sucrose) could be partially replaced by alternative sweeteners, including artificial LCS (sucralose), natural low-calorie bulk sweeteners (xylitol or erythritol), or rare sugars (D-allulose).

Physiological data provide experimental evidence that conventional sweeteners such as sucrose, glucose, and fructose elicit the secretion of GI satiation hormones while also suppressing the hunger hormone ghrelin (sucrose and glucose) and delaying gastric emptying rates. Glucose generally exhibits a more pronounced effect on GI hormones than do sucrose and fructose. In contrast, acute ingestion of artificial LCS like sucralose shows no effect on GI hormone secretion or gastric emptying rates. Notably, both xylitol and erythritol stimulate the release of GI hormones, slow down gastric emptying, and reduce subsequent energy intake. Meanwhile, the rare sugar D-allulose also induces the secretion of GI satiation hormones, but it does not affect gastric emptying rates. Stimulating gastrointestinal hormone release promotes satiation. Thus, using gastrointestinal hormone-stimulating sweeteners as preloads should reduce subsequent energy intake. However, comparing the effects of the sweeteners mentioned in this review on subsequent energy intake is challenging, due to variations in study design and heterogeneous outcomes, largely lacking data on total energy intake, and thus preventing definitive conclusions.

Conventional sweeteners have negative effects on glycemic control, blood lipids, and uric acid, and these effects vary according to the type of sweetener, dosage, and duration of administration. Glucose, for instance, triggers a rise in blood glucose concentrations with subsequent insulin secretion. This in turn stimulates glucose uptake, glycogenesis, and lipogenesis, and inhibits glycogenolysis [89,90,91], which means that energy reserves become accessible solely when insulin concentrations decrease again. Continuously elevated insulin concentrations, as seen with a high sugar consumption, encourage the development of adipose tissue and fatty liver disease. Conversely, fructose undergoes insulin-independent metabolism, passing from the small intestine to the liver through the portal vein, where it is converted into fatty acids [92,93]. Notably, substantial acute fructose intake has been observed to elevate the concentrations of blood lipids [65,94]. Furthermore, consistent consumption of significant fructose quantities is linked to non-alcoholic fatty liver disease (NAFLD) and is a factor contributing to the development of insulin resistance [66,95,96]. Additionally, consumption of sucrose or fructose increases plasma concentrations of uric acid, which can lead to gout and hypertension, and may contribute to the development of metabolic syndrome [97].

In contrast, acute sucralose consumption has no effect on glucose homeostasis [98,99]. However, when sucralose is taken in combination with other carbohydrates, which is a scenario closer to real-life conditions, the results are controversial. Therefore, more research is necessary to investigate the effects of the intake of chronic sucralose in combination with other carbohydrates on glucose homeostasis.

Xylitol and erythritol induce either a small rise or no rise at all in blood glucose and insulin concentrations after acute ingestion. Moreover, their chronic intake seems not to affect glucose tolerance and insulin resistance (HOMA-IR). Regarding blood lipids, most studies reviewed in this work found no changes in total cholesterol, LDL, HDL, or TG concentrations after ingestion of both polyols. Acute intake of xylitol induces a rise in uric acid concentrations, although a chronic intake had no effect. Neither acute nor chronic consumption of erythritol has been found to affect uric acid concentrations.

D-allulose seems to have positive effects on glycemic control. Indeed, some studies found that it reduces glucose and insulin concentrations during subsequent meals. Other studies found that it has no effect on glycemic control. Blood lipids and uric acid concentrations are not affected by D-allulose consumption.

## 10. Conclusions

Overall, in this review covering the metabolic effects of selected conventional and alternative sweeteners, several key observations have been made:

- Alternative sweeteners constitute a diverse group, and it would not be justified to extrapolate conclusions from the data on one substance to the cases of others. None of the mentioned sweeteners are inert; each exhibit one or more metabolic effects.
- Xylitol, erythritol, and D-allulose have promise as alternative sweeteners. They replicate the benefits of conventional sweeteners (e.g., sweetness and GI hormone release), while circumventing the detrimental effects of these substances on human health (e.g., blood glucose elevation or dyslipidemia).
- More randomized and controlled long-term interventional trials are necessary.

Some limitations of this review require consideration. This review only addresses the metabolic effects of a subset of the available sweeteners, and no conclusions can be drawn for other substances. The majority of the studies in this review concentrated on the acute effects of both conventional and alternative sweeteners on diverse metabolic outcomes. Further investigation is necessary to understand the implications of chronic exposure. Additionally, it is important to recognize that sweeteners may demonstrate varying behaviors when integrated into a food matrix with other nutrients or processed, considering factors such as heat, rather than when administered in isolation.

## Supplementary Materials

The following supporting information can be downloaded at: https://www.mdpi.com/article/10.3390/nu16050622/s1, Table S1: Effects on gastrointestinal hormones, Table S2: Effects on gastric emptying rates, Table S3: Effects on energy intake, Table S4: Effects on glycemic control, Table S5: Effects on blood lipids, Table S6: Effects on uric acid.
