# Supplementary material for: Metabolic Effects of Selected Conventional and Alternative Sweeteners: A Narrative Review
Source: Nutrients. 2024 Feb 23;16(5):622. doi: 10.3390/nu16050622 (PMC10933973; doi:10.3390/nu16050622)
Supplement: Supplementary file 1 [file nutrients-16-00622-s001.zip › nutrients-2789878-supplementary.pdf]

Supplementary Material

Table S1: Effects on gastrointestinal hormones

Name	Route and Dose of Administration	Duration of Administration	Participants	Observed Effects
Sucrose/glucose/fructose				
Ma et al. 2009, *American Journal of Physiology Gastrointestinal and Liver Physiology*	Intragastric administration of:<br>• 50 g sucrose in 500 mL saline<br>• 80 mg of sucralose in 500 mL saline, or<br>• 800 mg of sucralose in 500 mL saline, or<br>• 500 mL saline.	Acute	Healthy participants;<br>BMI mean: 21.6 ± 1.2 kg/m^2	Sucrose:<br>• ↑ GLP-1; ↑ GIP<br>Sucralose:<br>• No change in GLP-1 and GIP
Yunker et al. 2021, *JAMA Network Open*	Oral administration of:<br>• 75 g sucrose in 300 mL water, or<br>• 178 - 358 mg sucralose (individually matched) in 300 mL water, or<br>• 300 mL water.	Acute	Healthy participants;<br>BMI range: 19.2 - 40.3 kg/m^2	Sucrose:<br>• ↑ GLP-1<br>• No change in PYY<br>• ↓ Ghrelin<br>Sucralose:<br>• No change in GLP-1, PYY, and ghrelin
Tai et al. 2010 *Appetite*	Oral administration of:<br>• 100 g sucrose in 300 mL water, or<br>• 300 mL water.	Acute	Healthy participants;<br>BMI mean: 22.4 ± 0.5 kg/m^2	Sucrose:<br>• ↓ Ghrelin

Maersk et al. 2012<br>*European Journal of Clinical Nutrition*	Oral administration of:<br>• 53 g sucrose in 500 mL water (regular cola), or<br>• 23 g carbohydrates in 500 mL isocaloric semi-skimmed milk (carbohydrates 4.7 g/100 mL; protein 3.4 g/100 mL; fat 1.5 g/100 mL), or<br>• Aspartame in 500 mL water (diet cola), or<br>• 500 mL water.	Acute	Participants with overweight and obesity;<br>BMI range: 28 - 36 kg/m^2	Sucrose (regular cola):<br>• ↑ GLP-1; ↑ GIP<br>• ↓ Ghrelin<br>Semi-skimmed milk:<br>• ↑ GLP-1; ↑ GIP<br>• ↓ Ghrelin<br>Aspartame-sweetened diet cola:<br>• No change in GLP-1, GIP, and ghrelin<br><br>Comparison between treatments:<br>• Increase in GLP-1: semi-skimmed milk > sucrose<br>• Increase in GIP: semi-skimmed milk > sucrose<br>• Decrease in ghrelin: sucrose > semi-skimmed milk
Steinert et al. 2011<br>*British Journal of Nutrition*	Intragastric administration of:<br>• 50 g glucose in 250 mL water, or<br>• 25 g fructose in 250 mL water, or<br>• 62 mg sucralose in 250 mL water, or<br>• 169 mg aspartame in 250 mL water, or<br>• 220 mg acesulfame K in 250 mL water, or<br>• 250 mL water.	Acute	Healthy participants;<br>BMI range: 20.5 - 24.7 kg/m^2	Glucose:<br>• ↑ GLP-1; ↑ PYY<br>• ↓ Ghrelin<br>Fructose:<br>• No change in GLP-1, PYY, and ghrelin<br>Sucralose:<br>• No change in GLP-1, PYY, and ghrelin<br>Aspartame:<br>• No change in GLP-1, PYY, and ghrelin<br>Acesulfame K:<br>• No change in GLP-1, PYY, and ghrelin

Meyer-Gerspach et al. 2018<br>*American Journal of Clinical Nutrition*	Intragastric administration of:<br>• 50 g glucose in 250 mL water, or<br>• 25 g fructose in 250 mL water, or<br>• 220 mg acesulfame K in 250 mL water, or<br>• 250 mL water.	Acute	Healthy participants;<br>BMI range: 19 - 25 kg/m^2	Glucose:<br>• ↑ GLP-1; ↑ CCK<br>• ↓ Ghrelin<br>Fructose:<br>• ↑ GLP-1; ↑ CCK<br>• ↓ Ghrelin<br>Acesulfame K:<br>• No change in GLP-1, CCK, and ghrelin<br><br>Comparison between treatments:<br>• Increase in GLP-1: glucose = fructose<br>• Increase in CCK: glucose = fructose<br>• Decrease in ghrelin: glucose > fructose
Wölnerhanssen et al. 2015<br>*PLoS ONE*	Intragastric administration of:<br>• 75 g glucose in 300 mL water, or<br>• 25 g fructose in 300 mL water, or<br>• 300 mL water.	Acute	Healthy participants;<br>BMI range: 21.0 - 24.0 kg/m^2	Glucose:<br>• ↑ GLP-1; ↑ GIP<br>Fructose:<br>• ↑ GLP-1; ↑ GIP<br><br>Comparison between treatments:<br>• Increase in GLP-1: glucose > fructose<br>• Increase in GIP: glucose > fructose
Kong et al. 1999<br>*Peptides*	Oral administration of:<br>• 75 g glucose in 300 mL water, or<br>• 75 g fructose in 300 mL water, or<br>• 75 g glucose in 300 mL water followed by 75 g fructose in 300 mL 1h later.	Acute	Healthy participants;<br>BMI mean: 24.3 ± 2.9 kg/m^2	Glucose:<br>• ↑ GLP-1<br>Fructose:<br>• ↑ GLP-1<br>Glucose followed by fructose:<br>• No further increase in GLP-1 after fructose<br><br>Comparison between treatments:<br>• Increase in GLP-1: glucose > fructose

Yau et al. 2017<br>*Nutrients*	Oral administration of:<br>• 36 g sucrose in 600 mL water , or<br>• 39.6 g glucose in 600 mL water, or<br>• 36 g fructose in 600 mL water, or<br>• 19.8 g glucose & 18 g fructose in 600 mL water.	Acute	Healthy participants;<br>BMI mean: 25.5 ± 3.8 kg/m^2	Sucrose:<br>• ↑ GLP-1; ↑ GIP<br>• ↓ Ghrelin<br>Glucose:<br>• ↑ GLP-1; ↑ GIP<br>• ↓ Ghrelin<br>Fructose:<br>• ↑ GLP-1<br>• No change in GIP<br>• ↓ Ghrelin<br>Glucose & fructose:<br>• ↑ GLP-1; ↑ GIP<br>• ↓ Ghrelin<br><br>Comparison between treatments:<br>• Increase in GLP-1: sucrose = glucose = fructose = glucose & fructose<br>• Increase in GIP: glucose > glucose & fructose > sucrose<br>• Decrease in ghrelin: sucrose = glucose = fructose = glucose & fructose
Yunker et al. 2021<br>*The Journal of Clinical Endocrinology & Metabolism*	Oral administration of:<br>• 75 g sucrose in 300 mL water, or<br>• 75 g glucose in 300 mL water.	Acute	Healthy participants;<br>BMI mean: 27.0 ± 5.0 kg/m^2	Sucrose:<br>• ↑ GLP-1; ↑ PYY<br>• ↓ Ghrelin<br>Glucose:<br>• ↑ GLP-1; ↑ PYY<br>• ↓ Ghrelin<br><br>Comparison between treatments:<br>• Increase in GLP-1: sucrose < glucose<br>• Increase in PYY: sucrose < glucose<br>• Decrease in ghrelin: sucrose = glucose
Matikainen et al. 2017<br>*Nutrition, Metabolism & Cardiovascular Diseases*	Daily oral administration (3x/day) of:<br>• 25 g fructose in 330 mL water (total: 75 g/day)<br>OGTT and mixed meal test at baseline and after 12 weeks	Chronic (12 weeks)	Participants with overweight and obesity;<br>BMI range: 26.5-38.2 kg/m^2	Fructose:<br>• No change in fasting GLP-1, GIP, and PYY<br>• No change in GLP-1 and GIP during OGTT and mixed meal test<br>• No change in PYY during mixed meal

Sucralose				
Ma et al. 2009, *American Journal of Physiology Gastrointestinal and Liver Physiology*	Intragastric administration of:<br>• 80 mg sucralose in 500 mL saline, or<br>• 800 mg sucralose in 500 mL saline, or<br>• 50 g sucrose in 500 mL saline, or<br>• 500 mL saline.	Acute	Healthy participants;<br>BMI mean: 21.6 ± 1.2 kg/m^2	Sucralose:<br>• No change in GLP-1 and GIP<br>Sucrose:<br>• ↑ GLP-1; ↑ GIP
Yunker et al. 2021, *JAMA Network Open*	Oral administration of:<br>• 178 - 358 mg sucralose (individually matched) in 300 mL water, or<br>• 75 g sucrose in 300 mL water, or<br>• 300 mL water.	Acute	Healthy participants;<br>BMI range: 19.2 - 40.3 kg/m^2	Sucralose:<br>• No change in GLP-1, PYY, and ghrelin<br>Sucrose:<br>• ↑ GLP-1<br>• No change in PYY<br>• ↓ Ghrelin
Steinert et al. 2011 *British Journal of Nutrition*	Intragastric administration of:<br>• 62 mg sucralose in 250 mL water, or<br>• 50 g glucose in 250 mL water, or<br>• 25 g fructose in 250 mL water, or<br>• 169 mg aspartame in 250 mL water, or<br>• 220 mg acesulfame K in 250 mL water, or<br>• 250 mL water.	Acute	Healthy participants;<br>BMI mean: 23.0 ± 0.5 kg/m^2	Sucralose:<br>• No change in GLP-1, PYY, and ghrelin<br>Glucose:<br>• ↑ GLP-1; ↑ PYY<br>• ↓ Ghrelin<br>Fructose:<br>• No change in GLP-1, PYY, and ghrelin<br>Aspartame:<br>• No change in GLP-1, PYY, and ghrelin<br>Acesulfame K:<br>• No change in GLP-1, PYY, and ghrelin
Sylvetsky et al. 2016 *Nutrition & Metabolism*	Oral administration of preloads with:<br>• 68 mg sucralose in 355 mL water, or<br>• 170 mg sucralose in 355 mL water, or<br>• 250 mg sucralose in 355 mL water, or<br>• 355 mL water<br>followed by an OGTT.	Acute	Healthy participants;<br>BMI mean: 25.8 ± 4.2 kg/m^2	Sucralose:<br>• No change in GLP-1, and GIP after preload<br><br>Comparison between treatments:<br>• Increase in GLP-1 and GIP during OGTT: sucralose (all concentrations) = water

Wu et al. 2013<br>*Diabetes Care*	Oral administration of preloads with:<br>• 52 mg sucralose in 240 mL water, or<br>• 200 mg acesulfame K in 240 mL water, or<br>• 46 mg sucralose & 26 mg acesulfame K in 240 mL water, or<br>• 240 mL water<br>followed by an OGTT.	Acute	Healthy participants;<br>BMI mean: 25.5 ± 1.0 kg/m^2	Sucralose:<br>• No change in GLP-1 after preload<br>Acesulfame K:<br>• No change in GLP-1 after preload<br>Sucralose & acesulfame K:<br>• No change in GLP-1 after preload<br><br>Comparison between treatments:<br>• Increase in GLP-1 during OGTT: sucralose = acesulfame K = sucralose & acesulfame K
Wu et al. 2012<br>*American Journal of Clinical Nutrition*	Oral administration of preloads with:<br>• 60 mg sucralose in 400 mL water, or<br>• 40 g glucose in 400 mL water, or<br>• 40 g 3-O-methyl-glucose in 400 mL water, or<br>• 40 g tagatose & isomalt in 400 mL water<br>followed by a labeled mashed potato meal containing ^{13}C octanoic acid.	Acute	Healthy participants;<br>BMI mean: 25.5 ± 1.5 kg/m2	Sucralose:<br>• No change in GLP-1 and GIP after preload<br>Glucose:<br>• ↑ GLP-1; ↑ GIP after preload<br>3-O-methyl-glucose:<br>• ↑ GLP-1; ↑ GIP after preload<br>Tagatose & isomalt:<br>• No change in GLP-1 and GIP after preload<br><br>Comparison between treatments:<br>• Increase in GLP-1 after preload: glucose < 3-OMG<br>• Increase in GIP after preload: glucose > 3-OMG<br>• Increase in GLP-1 during test meal: sucralose < glucose or 3-OMG or tagatose & isomalt<br>• Increase in GIP during test meal: sucralose or tagatose & isomalt < glucose or 3-OMG
Ma et al. 2010<br>*British Journal of Nutrition*	Intraduodenal administration of:<br>• 960 mg sucralose in 600 mL saline, or<br>• 600 mL saline.	Acute	Healthy participants;<br>BMI mean: 23.4 ± 0.8 kg/m^2	Sucralose:<br>• No change in GLP-1

Ford et al. 2011<br>*European Journal of Clinical Nutrition*	Oral administration of:<br>• 4.15 mg sucralose in 50 mL water (i.e. 0.083% sucralose solution), or<br>• 4.15 mg sucralose & 25 g maltodextrin in 50 mL water (i.e. 0.083% sucralose solution & 50% maltodextrin solution), or<br>• 50 mL water.	Acute	Healthy participants;<br>BMI range: 18.0 - 23.9 kg/m^2	Sucralose:<br>• No change in GLP-1
Brown et al. 2012<br>*Diabetes Care*	Oral administration of preloads with:<br>• 45.6 mg sucralose in 240 mL diet soda (mean concentration of sucralose: 190 ± 38 µg/mL), or<br>• 240 mL carbonated water,<br>followed by an OGTT.	Acute	Healthy participants;<br>BMI range: 19.1 - 35.9 kg/m^2	Comparison between treatments:<br>• Increase in GLP-1 during OGTT: sucralose > water<br>• Increase in GIP and PYY during OGTT: sucralose = water
Temizkan et al. 2015<br>*European Journal of Clinical Nutrition*	Oral administration of preloads with:<br>• 24 mg sucralose in 200 mL water, or<br>• 200 mL water,<br>followed by an OGTT.	Acute	Healthy participants;<br>BMI mean: 30.3 ± 4.5 kg/m^2	Comparison between treatments:<br>• Increase in GLP-1 during OGTT: sucralose > water
Suez et al. 2022<br>*Cell*	Daily oral administration (3x/day) of:<br>• 204 mg sucralose (total: 612 mg/day) & 11.796 g glucose as a bulking agent (total: 35.388 g/day), or<br>• 11.796 g glucose alone (total: 35.388 g/day).<br>OGTT at baseline and after 2 weeks.	Chronic (2 weeks)	Healthy participants;<br>BMI range: 18 - 28 kg/m^2	Sucralose & glucose:<br>• No change in non-fasting GLP-1 at baseline, during treatment, and follow-up<br>Glucose:<br>• No change in non-fasting GLP-1 at baseline, during treatment, and follow-up
Lertrit et al. 2018<br>*Nutrition*	Daily oral administration (1x/day) of:<br>• 200 mg sucralose in capsules, or<br>• empty capsules (placebo),<br>OGTT, or IVGTT at baseline and after 4 weeks.	Chronic (4 weeks)	Healthy participants;<br>BMI range: 18.5 – 27.0 kg/m^2	Comparison between treatments:<br>• Increase in GLP-1 during OGTT: sucralose capsules > empty capsules
Xylitol				

Meyer-Gerspach et al. 2021 *Nutrients*	Intragastric administration of:<br>• 7 g xylitol in 300 mL water, or<br>• 17 g xylitol in 300 mL water, or<br>• 35 g xylitol in 300 mL water.	Acute	Healthy participants; BMI range 19.4 - 23.0 kg/m^2	Xylitol:<br>• Dose-dependent ↑ GLP-1, dose-dependent ↑ PYY<br>• No change in GIP<br>• Dose-dependent ↑ CCK
Meyer-Gerspach et al. 2022 *Nutritional Neuroscience*	Intragastric administration of:<br>• 50 g xylitol in 300 mL water.	Acute	Healthy participants; BMI range: 20.0 - 38.9 kg/m^2	Xylitol:<br>• ↑ PYY; ↑ CCK
Wölnerhanssen et al. 2016 *American Journal of Physiology Endocrinology and Metabolism*	Intragastric administration of:<br>• 50 g xylitol in 300 mL water.	Acute	Healthy participants and participants with obesity; BMI range 19.9 - 48.2 kg/m^2	Xylitol:<br>• ↑ GLP-1; ↑ CCK
Erythritol				
Meyer-Gerspach et al. 2022 *Nutritional Neuroscience*	Intragastric administration of:<br>• 75 g erythritol in 300 mL water.	Acute	Healthy participants; BMI range: 20.0 - 38.9 kg/m^2	Erythritol:<br>• ↑ PYY; ↑ CCK
Wölnerhanssen et al. 2016 *American Journal of Physiology Endocrinology and Metabolism*	Intragastric administration of:<br>• 75 g erythritol in 300 mL water.	Acute	Healthy participants and participants with obesity; BMI range: 19.9 - 48.2 kg/m^2	Erythritol:<br>• ↑ GLP-1; ↑ CCK
Wölnerhanssen et al. 2021 *Diabetes, Obesity and Metabolism*	Intragastric administration of:<br>• 10 g erythritol in 300 mL water, or<br>• 20 g erythritol in 300 mL water, or<br>• 50 g erythritol in 300 mL water, or<br>• 300 mL water.	Acute	Healthy participants; BMI range: 19.4 - 24.0 kg/m^2	Erythritol:<br>• Dose-dependent ↑ GLP-1; dose-dependent ↑ PYY<br>• No change in GIP<br>• Dose-dependent ↑ CCK

Teysseire et al. 2022<br>*Nutrients*	Oral administration of preloads with:<br>• 50 g erythritol in 300 mL water, or<br>• 33.5 g sucrose in 300 mL water, or<br>• 55.8 mg sucralose in 300 mL water, or<br>• 300 mL water,<br>subsequent *ad libitum* a test meal.	Acute	Healthy participants;<br>BMI range: 19.6 - 24.8 kg/m^2	Erythritol:<br>• ↑ CCK after preload<br>Sucrose:<br>• ↑ CCK after preload<br>Sucralose:<br>• No change in CCK after preload<br><br>Comparison between treatments:<br>• Increase in CCK after preload: erythritol > sucrose<br>• Increase in CCK during test meal: erythritol > sucrose, sucralose
Teysseire et al. 2022<br>*The Journal of Nutrition*	Intragastric administration of:<br>• 50 g erythritol in 300 mL water, or<br>• 25 g D-allulose in 300 mL water, or<br>• 300 mL water.	Acute	Healthy participants;<br>BMI range: 19.1 - 24.3 kg/m^2	Erythritol:<br>• ↑ GLP-1; ↑ PYY; ↑ CCK<br>D-allulose:<br>• ↑ GLP-1; ↑ PYY; ↑ CCK
Overduin et al. 2016<br>*Appetite*	Oral administration of breakfasts containing skimmed milk and starch with:<br>• 10% sucrose, or<br>• 8% erythritol & 2% sucrose (isovolumic to sucrose breakfast), or<br>• 8% erythritol & 2% sucrose (isocaloric to sucrose breakfast)<br>subsequent *ad libitum* buffet meal.	Acute	• Healthy participants; BMI mean: 21.8 ± 2.0 kg/m^2<br>• Participants with obesity; BMI mean: 34.2 ± 3.6 kg/m^2	Erythritol & sucrose breakfast:<br>• ↑ GLP-1; ↑ PYY after breakfast preload<br>Sucrose breakfast:<br>• ↑ GLP-1; ↑ PYY after breakfast preload<br><br>Comparison between treatments:<br>• Increase in GLP-1 and PYY after breakfast preload: erythritol & sucrose breakfast = sucrose breakfast
Teysseire et al. 2023<br>*Nutrients*	Intragastric administration of:<br>• 50 g erythritol in 300 mL water, or<br>• 25 g D-allulose in 300 mL water, or<br>• 300 mL water.	Acute	Healthy participants;<br>BMI range: 19.1 - 24.3 kg/m^2	Erythritol:<br>• ↓ Ghrelin<br>D-allulose:<br>• No change in ghrelin
Sorrentino et al. 2020<br>*Cureus*	Oral administration of:<br>• 50.8 g erythritol in 250 mL water, or<br>• 185 mg aspartame in 250 mL water.	Acute	Healthy participants;<br>BMI mean: 21.7 ± 1.9 kg/m^2	Erythritol:<br>• ↓ Ghrelin<br>Aspartame:<br>• No change in ghrelin
D-allulose				

Teysseire et al. 2022<br>*The Journal of Nutrition*	Intragastric administration of:<br>• 25 g D-allulose in 300 mL water, or<br>• 50 g erythritol in 300 mL water, or<br>• 300 mL water.	Acute	Healthy participants;<br>BMI range: 19.1 - 24.3 kg/m^2	D-allulose:<br>• ↑ GLP-1; ↑ PYY; ↑ CCK<br>Erythritol:<br>• ↑ GLP-1; ↑ PYY; ↑ CCK
Teysseire et al. 2023<br>*Nutrients*	Intragastric administration of:<br>• 25 g D-allulose in 300 mL water, or<br>• 50 g erythritol in 300 mL water, or<br>• 300 mL water.	Acute	Healthy participants;<br>BMI range: 19.1 - 24.3 kg/m^2	D-allulose:<br>• No change in ghrelin<br>Erythritol:<br>• ↓ Ghrelin

Table S2: Effects on gastric emptying rates

Name	Route and Dose of Administration	Duration of Administration	Participants	Observed Effects
Sucrose/glucose/fructose				
Ma et al. 2009, *American Journal of Physiology Gastrointestinal and Liver Physiology*	Intragastric administration of:<br>• 50 g sucrose in 500 mL saline, or<br>• 80 mg of sucralose in 500 mL saline, or<br>• 800 mg of sucralose in 500 mL saline, or<br>• 500 mL saline.	Acute	Healthy participants;<br>BMI mean: 21.6 ± 1.2 kg/m2	Comparison between treatments:<br>• Retardation of gastric emptying sucrose > sucralose = saline
Tai et al. 2010 *Appetite*	Oral administration of:<br>• 100 g sucrose in 300 mL water, or<br>• 300 mL water.	Acute	Healthy participants;<br>BMI mean: 22.4 ± 0.5 kg/m^2	Comparison between treatments:<br>• Retardation of gastric emptying: sucrose > water
Lavin et al. 2002 *International Journal of Obesity*	Oral administration of:<br>• 125 g sucrose in 450 mL water & 50 mL lemon juice, or<br>• 525 mL water & 50 mL lemon juice.	Acute	Healthy participants;<br>BMI range 20.1 - 24.7 kg/m2	Comparison between treatments:<br>• Retardation of gastric emptying: sucrose & lemon juice > water & lemon juice
Brener et al. 1983 *Gastroenterology*	Oral administration of:<br>• 20 g glucose in 400 mL water, or<br>• 50 g glucose in 400 mL water, or<br>• 100 g glucose in 400 mL water, or<br>• 400 mL saline.	Acute	Healthy participants;<br>Normal weight	Comparison between treatments:<br>• Retardation of gastric emptying: glucose (dose-dependent) > saline
Guss et al. 1994 *American Journal of Physiology*	Oral administration of solutions containing 15 mL lemon juice and 147 mg aspartame with:<br>• 50 g glucose in 500 mL water (i.e. 10% glucose solution), or<br>• 50 g fructose in 500 mL water (i.e. 10% fructose solution), or<br>• 500 mL water.	Acute	Healthy participants;<br>BMI mean: 19.9 ± 0.6 kg/m2	Comparison between treatments:<br>• Retardation of gastric emptying: glucose > fructose > water
Shi et al. 2017 *Medicine & Science in Sports & Exercise*	Oral administration of 7 mL/kg body weight (corresponding to 490 mL for a body weight of 70kg) of:<br>• 9.8 g sucrose or glucose in 490 mL water (i.e. 2% sucrose or glucose solution), or	Acute	Healthy participants;<br>Body weight mean: 69.2 ± 10.6 kg	Comparison between treatments:<br>• Retardation of gastric emptying: glucose > sucrose > water

	• 19.6 g sucrose or glucose in 490 mL water (i.e. 4% sucrose or glucose solution), or<br>• 29.4 g sucrose or glucose in 490 mL water (i.e. 6% sucrose or glucose solution), or<br>• 39.4 g sucrose or glucose in 490 mL water (i.e. 8% sucrose or glucose solution), or<br>• 490 mL water.			
Horowitz et al. 1996<br>*Diabetologia*	Acute oral administration of glucose or fructose solution with and without prior glucose supplementation (4-7 days with 440 g/day of glucose):<br>• 75 g glucose in 350 mL water (without prior glucose supplementation), or<br>• 75 g glucose in 350 mL water (with prior glucose supplementation), or<br>• 75 g fructose in 350 mL water (without prior glucose supplementation), or<br>• 75 g fructose in 350 mL water (with prior glucose supplementation).	Acute/Chronic	Healthy participants;<br>Body weight range: 50 - 91 kg	Comparison between chronic treatments (with *vs.* without prior glucose supplementation):<br>• Acceleration of gastric emptying of both fructose or glucose solutions: with prior glucose supplementation > without prior glucose supplementation
Yau et al. 2014<br>*Nutrition*<br>Yau et al. 2017<br>*Nutrients*	Acute oral administration of fructose or glucose solution with and without prior fructose supplementation (3 days with 120 g/day of fructose):<br>• 39.6 g glucose in 600 mL water (without prior fructose supplementation), or<br>• 39.6 g glucose in 600 mL water (with prior fructose supplementation), or<br>• 36 g fructose in 600 mL water (without prior fructose supplementation), or<br>• 36 g fructose in 600 mL water (with prior fructose supplementation).	Acute/Chronic	Healthy participants;<br>BMI mean: 25.3 ± 3.1 kg/m^2	Comparison between chronic treatments (with *vs.* without prior fructose supplementation):<br>• Acceleration of gastric emptying of glucose solution: with prior fructose supplementation = without prior fructose supplementation<br>• Acceleration of gastric emptying of fructose solution: with prior fructose supplementation > without prior fructose supplementation
Sucralose				

Sylvetsky et al. 2016<br>*Nutrition & Metabolism*	Oral administration of preloads with:<br>• 68 g sucralose in 355 mL water, or<br>• 170 g sucralose in 355 mL water, or<br>• 250 g sucralose in 355 mL water, or<br>• 355 mL water,<br>followed by OGTT.	Acute	Healthy participants;<br>BMI mean: 25.8 ± 4.2 kg/m^2	Comparison between treatments:<br>• Retardation of gastric emptying during OGTT: sucralose = water
Ma et al. 2009,<br>*American Journal of Physiology Gastrointestinal and Liver Physiology*	Intragastric administration of:<br>• 80 mg sucralose in 500 mL saline, or<br>• 800 mg sucralose in 500 mL saline, or<br>• 50 g sucrose in 500 mL saline, or<br>• 500 mL saline	Acute	Healthy participants;<br>BMI mean: 21.6 ± 1.2 kg/m2	Comparison between treatments:<br>• Retardation of gastric emptying: sucralose = saline < sucrose
Wu et al. 2012<br>*American Journal of Clinical Nutrition*	Oral administration of preloads with:<br>• 60 mg sucralose in 400 mL water, or<br>• 40 g glucose in 400 mL water, or<br>• 40 g 3-O-methyl-glucose in 400 mL water, or<br>• 40 g tagatose & isomalt in 400 mL water,<br>followed by a labeled mashed potato meal containing ^{13}C octanoic acid.	Acute	Healthy participants;<br>BMI mean: 25.5 ± 1.5 kg/m^2	Comparison between treatments:<br>• Retardation of gastric emptying of mashed potato meal: sucralose = glucose;<br>sucralose < 3-O-methyl-glucose, or tagatose & isomalt;<br>glucose = 3-O-methyl-glucose = tagatose & isomalt
Xylitol				
Meyer-Gerspach et al. 2021<br>*Nutrients*	Intragastric administration of:<br>• 7 g xylitol in 300 mL water, or<br>• 17 g xylitol in 300 mL water, or<br>• 35 g xylitol in 300 mL water, or<br>• 300 mL water.	Acute	Healthy participants;<br>BMI range 19.4 - 23.0 kg/m^2	Comparison of treatments:<br>• Retardation of gastric emptying: xylitol (dose-dependent) > water
Wölnerhanssen et al. 2016<br>*American Journal of Physiology Endocrinology and Metabolism*	Intragastric administration of:<br>• 50 g xylitol in 300 mL water, or<br>• 300 mL water.	Acute	Healthy participants and participants with obesity;<br>BMI range 19.9 - 48.2 kg/m^2	Comparison between treatments:<br>• Retardation of gastric emptying: xylitol > water

Shafer et al. 1987<br>*American Journal of Clinical Nutrition*	Oral administration of:<br>• 25 g xylitol in 50 mL water, or<br>• 25 g glucose in 50 mL water, or<br>• 50 mL water<br>together with radio-labeled scrambled eggs.	Acute	Healthy participants	Comparison between treatmens:<br>• Retardation of gastric emptying: xylitol > glucose > water
Erythritol				
Wölnerhanssen et al. 2016<br>*American Journal of Physiology Endocrinology and Metabolism*	Intragastric administration of:<br>• 75 g erythritol in 300 mL water, or<br>• 300 mL water.	Acute	Healthy participants and participants with obesity; BMI range 19.9 - 48.2 kg/m^2	Comparison between treatments:<br>• Retardation of gastric emptying: erythritol > water
Wölnerhanssen et al. 2021<br>*Diabetes, Obesity and Metabolism*	Intragastric administration of:<br>• 10 g erythritol in 300 mL water, or<br>• 20 g erythritol in 300 mL water, or<br>• 50 g erythritol in 300 mL water, or<br>• 300 mL water.	Acute	Healthy participants; BMI range: 19.4 - 24.0 kg/m^2	Comparison between treatments:<br>• Retardation of gastric emptying: erythritol (dose-dependent) > water
Teysseire et al. 2022<br>*The Journal of Nutrition*	Intragastric administration of:<br>• 50 g erythritol in 300 mL water, or<br>• 25 g D-allulose in 300 mL water, or<br>• 300 mL water.	Acute	Healthy participants; BMI range: 19.1 - 24.3 kg/m^2	Comparison between treatments:<br>• Retardation of gastric emptying: erythritol > D-allulose = water
D-allulose				
Teysseire et al. 2022<br>*The Journal of Nutrition*	Intragastric administration of:<br>• 25 g D-allulose in 300 mL water, or<br>• 50 g erythritol in 300 mL water, or<br>• 300 mL water.	Acute	Healthy participants; BMI range: 19.1 - 24.3 kg/m^2	Comparison between treatments: D-allulose:<br>• Retardation of gastric emptying: D-allulose = water < erythritol

Table S3: Effects on energy intake

Name	Route and Dose of Administration	Duration of Administration	Participants	Observed Effects
Sucrose/glucose/fructose				
Farhat et al. 2019<br>*Nutrients*	Oral administration of preloads with:<br>• 60 g sucrose in 300 mL water, or<br>• 300 mL water,<br>subsequent *ad libitum* test meal.	Acute	Healthy participants;<br>BMI mean: 23.4 ± 3.4 kg/m^2	Comparison between treatments:<br>• Subsequent energy intake: sucrose = water preloads
Rolls et al. 1990<br>*Physiology & Behavior*	Oral administration of:<br>• 20 g sucrose in 236.59 mL lemonade, or<br>• 40 g sucrose in 473.18 mL lemonade, or<br>• 0.11 mg aspartame in 236.59 mL lemonade, or<br>• 220 mg aspartame in 473.18 mL lemonade, or<br>• 236.59 mL water, or<br>• 473.18 mL water<br>simultaneous *ad libitum* test meal.	Acute	Healthy participants	Comparison between treatments:<br>• Food intake (only test meal): sucrose = aspartame = water<br>• Total energy intake (drinks & test meal): sucrose > aspartame or water
Melanson et al. 2007<br>*Nutrition*	Oral administration of:<br>• 134 g sucrose in a beverage (corresponding to 30% of total daily energy intake (over 24 h)), or<br>• 192 g of HFCS in a beverage (corresponding to 30% of total daily energy intake (over 24 h))<br>assessment of total daily energy intake (over 24 h) on the day after ingestion.	Acute	Healthy participants;<br>BMI mean: 22.4 ± 1.7 kg/m^2	Comparison between treatments:<br>• Total energy intake: sucrose = HFCS
Tey et al. 2017<br>*International Journal of Obesity*	Oral administration of preloads with:<br>• 65 g sucrose in 500 mL water, or<br>• 440 mg aspartame in 500 mL water, or<br>• 0.63 g monk fruit in 500 mL water, or<br>• 0.33 g stevia in 500 mL water,<br>subsequent *ad libitum* test meal.	Acute	Healthy participants;<br>BMI mean: 21.7 ± 1.9 kg/m^2	Comparison between treatments:<br>• Subsequent energy intake: sucrose < aspartame, monk fruit, or stevia preloads<br>• Total energy intake: sucrose = aspartame = monk fruit = stevia preloads

Crézé et al. 2018 *Nutrients*	Oral administration of a first preload together with a standardized meal at 0 min and a second preload at 210 min followed by a buffet meal:<br><br>Preloads at 0 min:<br>• 37.1 g sucrose in 350 mL water, or<br>• 137.2 mg cyclamate, 63.35 mg acesulfame K, & 40.6 mg aspartame in 350 mL water (LCS), or<br>• 350 mL water<br>together with a standardized meal.<br><br>Preloads at 210 min:<br>• 21.2 g sucrose in 350 mL water, or<br>• 78.4 mg cyclamate, 36.2 mg acesulfame K, & 23.2 mg aspartame in 350 mL water (LCS), or<br>• 350 mL water.<br>subsequent *ad libitum* buffet meal.	Acute	Healthy participants; BMI mean: 21.4 ± 0.4 kg/m^2	Comparison between treatments:<br>• Total energy intake: sucrose < LCS or water preloads
Yunker et al. 2021, *JAMA Network Open*	Oral administration of preloads with:<br>• 75 g sucrose in 300 mL water, or<br>• 178 - 358 mg sucralose (individually matched) in 300 mL water, or<br>• 300 mL water,<br>subsequent *ad libitum* test meal.	Acute	Healthy participants; BMI range: 19.2 - 40.3 kg/m^2	Comparison between treatments:<br>• Total energy intake: sucrose < sucralose preloads
Guss et al. 1994 *American Journal of Physiology*	Oral administration of preloads containing 15 mL lemon juice and 147 mg aspartame together with:<br>• 5 g glucose in 500 mL water (i.e. 1% glucose solution), or<br>• 5 g fructose in 500 mL water (i.e. 1% fructose solution), or<br>• 50 g glucose in 500 mL water (i.e. 10% glucose solution), or	Acute	Healthy participants; BMI mean: 21.4 ± 1.0 kg/m2	Comparison between treatments:<br>• Subsequent energy intake group 1 (testmeal at 30 min): 1% glucose or fructose solutions (mean of 75.8 g) > water preloads; 10% glucose or fructose solutions (mean of 52.2 g, not significant) < water preloads; glucose = fructose preloads<br>• Subsequenet energy intake group 2 (testmeal at 135 min): glucose = fructose preloads

	• 50 g fructose in 500 mL water (i.e. 10% fructose solution), or<br>• 500 mL water<br>subsequent *ad libitum* test meal at 30 min (group 1), and 135 min (group 2) after the preloads.			• Total energy intake: 10% fructose or 1% glucose solution < water preloads
Maersk et al. 2012<br>*American Journal of Clinical Nutrition*	Daily oral daily administration of:<br>• 106 g sucrose in 1000 mL water (regular cola), or<br>• 47 g carbohydrates in 1000 mL isocaloric semi-skimmed milk (carbohydrates 4.7 g/100 mL; protein 3.4 g/100 mL; fat 1.5 g/100 mL), or<br>• aspartame-sweetened diet cola (1000 mL), or<br>• 1000 mL water	Chronic (6 months)	Participants with overweight and obesity;<br>BMI range: 26 - 40 kg/m^2	Comparison between treatments:<br>• Total energy intake: sucrose = semi-skimmed milk = aspartame-sweetened diet cola = water
Kuzma et al. 2015<br>*American Journal of Clinical Nutrition (Study A)*	Daily oral administration (4x/day) of:<br>• glucose (corresponding to 25% of daily energy requirement), or<br>• fructose (corresponding to 25% of daily energy requirement), or<br>• aspartame	Chronic (8 days)	Healthy participants;<br>BMI mean: 22.7 ± 1.3 kg/m^2	Ccomparison between treatments:<br>• Total Energy intake: glucose = fructose > aspartame
Kuzma et al. 2015<br>*American Journal of Clinical Nutrition (Study B)*	Daily oral administration (4x/day) of:<br>• glucose (correspondint to 25% of daily energy requirement), or<br>• fructose (corresponding to 25% of daily energy requirement), or<br>• HFCS (55% fructose, 41% glucose, and 4% higher saccharides)	Chronic (8 days)	Healthy participants and participants with overweight and obesity;<br>BMI mean: 27.4 ± 4.8 kg/m^2	Comparison between treatments:<br>• Total energy intake: glucose = fructose = HFCS
Sigala et al.2020<br>*Nutrients*	Daily oral administration (3x/day) of:<br>• glucose (corresponding to 25% of daily energy requirement), or<br>• fructose (corresponding to 25% of daily energy requirement), or,<br>• sucrose (corresponding to 25% of daily energy requirement), or	Chronic (2 weeks)	Healthy participants;<br>BMI mean: 25.8 ± 3.5 kg/m^2 (glucose), 25.4 ± 3.7 kg/m^2 (fructose), 25.3 ± 3.4 kg/m^2 (sucrose), 24.9 ± 3.9 kg/m^2 (HFCS), 24.8 ± 3.3 kg/m^2 (aspartame)	Comparison between treatments:<br>• Total energy intake: glucose =fructose = sucrose = HFCS > aspartame

	• HFCS (providing 25% of daily energy requirement), or<br>• aspartame<br>*ad libitum* test meals (breakfast, lunch, dinner) at week 0 and week 2			
Geidl et al. 2021<br>*Journal of Hepatology*	Daily oral administration (3x/day) of:<br>• 26.6 g sucrose in 200 mL water (total: 79.8 g/day)<br>• 26.6 g glucose in 200 mL water (total: 79.8 g/day)<br>• 26.6 g fructose in 200 mL water (total: 79.8 g/day)<br>• No beverage<br>OGTT at baseline and after 7 weeks.	Chronic (7weeks)	Healthy participants;<br>BMI mean: 21.8 ± 1.6 kg/m²	Comparison between treatments:<br>• Total energy intake: sucrose = glucose = fructose
Reid et al. 2007<br>*British Journal of Nutrition*	Daily oral administration (4x/day) of:<br>• 21 g sucrose in 250 mL water (Irn-Bru)<br>• 1.78 aspartame in 250 mL water (diet Irn-Bru)	Chronic (4 weeks)	Healthy participants;<br>BMI mean: 22.5 ± 2.8 kg/m²	Comparison between treatments:<br>• Total energy intake: sucrose = aspartame
Sucralose				
Ford et al. 2011<br>*European Journal of Clinical Nutrition*	Oral administration of preloads with:<br>• 4.15 mg sucralose in 50 mL water (i.e. 0.083% sucralose solution), or<br>• 4.15 mg sucralose & 25 g maltodextrin in 50 mL water (i.e. 0.083% sucralose solution & 50% maltodextrin solution), or<br>• 50 mL water<br>subsequent *ad libitum* test meal.	Acute	Healthy participants;<br>BMI range: 18.8 - 23.9 kg/m²	Comparison between treatments:<br>• Subsequent energy intake: sucralose = water preloads
Gadah et al. 2016<br>*Appetite*	The study tested the difference between a crossover and parallel-group study design on energy intake.<br>Oral administration of preloads with:<br>• 0.7 g sucralose in 250 mL water & 50 mL blackcurrant squash juice, 0.36 g	Acute	Healthy participants;<br>BMI mean: 22.9 ± 3.2 kg/m2	Comparison between treatments:<br>• Subsequent energy intake: sucralose > sucrose preloads (crossover design); sucralose > sucrose preloads (parallel design)

	thickening agent → 300 mL serving volume, or<br>• 41.3 g sucrose in 211 mL water & 89 mL blackcurrant squash jucie → 300 mL serving volume, or<br>• 300 mL water,<br>subsequent *ad libitum* test meal.			• Total energy intake: sucralose preloads (crossover design) < sucrose; sucralose preloads (parallel-design) ≤ sucrose
Akhavan et al. 2011<br>*International Journal of Obesity*	Oral administration of preloads with:<br>• 130 mg sucralose in 300 mL water, or<br>• 75 g sucrose in 300 mL water & lemon concentrate, or<br>• 37.5 g glucose & 37.5 g fructose in 300 mL water (i.e. 50% glucose & 50% fructose solutions),<br>subsequent *ad libitum* test meal.	Acute	Healthy participants<br>BMI mean: 22.1 ± 0.5 kg/m²	Comparison between treatments:<br>• Subsequent energy intake: sucralose = glucose & fructose = sucrose preloads<br>• Total energy intake: sucralose < sucrose or glucose & fructose preloads
Chern and Tan 2019<br>*Nutrients*	Oral administration of solid preloads (jelly) with:<br>• 120 mg of sucralose, or<br>• 50 g of sucrose, or<br>• 120 mg of sucralose & maltodextrin matched in carbohydrate content with 50 g sucrose,<br>subsequent *ad libitum* test meal.	Acute	Healthy participants and participants with overweight;<br>BMI mean: 25.0 ± 4.7 kg/m²	Comparison between treatments:<br>• Total energy intake: sucralose > sucrose or sucralose & maltodextrin preloads
Yunker et al. 2021,<br>*JAMA Network Open*	Oral administration of preloads with:<br>• 178 - 358 mg sucralose (individually matched) in 300 mL water, or<br>• 75 g sucrose in 300 mL water, or<br>• 300 mL water,<br>subsequent *ad libitum* test meal.	Acute	Healthy participants;<br>BMI range: 19.2 - 40.3 kg/m²	Comparison between treatments:<br>• Total energy intake: sucralose > sucrose preloads
Higgins and Mattes 2019<br>*American Journal of Clinical Nutrition*	Daily oral administration of:<br>• 160 mg of sucralose, or<br>• 730 mg of saccharin, or<br>• 580 mg of aspartame, or<br>• 660 mg of rebaudioside A, or	Chronic (12 weeks)	Participants with overweight and obesity;<br>BMI range: 25 - 40 kg/m²	Comparison between treatments:<br>• Total energy intake: sucrose > aspartame or sucralose or saccharin or rebaudioside A

	• 100, 120, or 140 g of sucrose,<br>• In 1.25, 1.5, or 1.75 L water/day (volume depending on body weight)			
Xylitol				
Shafer et al. 1987<br>*American Journal of Clinical Nutrition*	Oral administration of preloads with:<br>• 25 g of xylitol in 50 mL water, or<br>• 50 mL water,<br>subsequent *ad libitum* test meal.	Acute	Healthy participants	Comparison between treatments:<br>• Subsequent energy intake: xylitol < water preloads
Flad et al.<br>*unpublished*	Oral administration of preloads with:<br>• 33.5 g xylitol in 300 mL water, or<br>• 33.5 g sucrose in 300 mL water, or<br>• 167.5 mg acesulfame K in 300 mL water, or<br>• 300 mL water,<br>subsequent *ad libitum* test meal.	Acute	Healthy participants;<br>BMI range 19.9 - 24.7 kg/m^2	Comparison between treatments:<br>• Total energy intake: xylitol or acesulfame K < sucrose preloads
Erythritol				
Overduin et al. 2016<br>*Appetite*	Oral administration of breakfasts containing skimmed milk and starch with:<br>• 10% sucrose, or<br>• 8% erythritol & 2% sucrose (isovolumic to sucrose breakfast), or<br>• 8% erythritol & 2% sucrose (isocaloric to sucrose breakfast)<br>subsequent *ad libitum* buffet meal.	Acute	• Healthy participants; BMI mean: 21.8 ± 2.0 kg/m^2<br>• Participants with obesity; BMI mean: 34.2 ± 3.6 kg/m^2	Comparison between treatments:<br>• Subsequent energy intake: erythritol & sucrose = sucrose
Teysseire et al 2022<br>*Nutrients*	Oral administration of preloads with:<br>• 50 g of erythritol in 300 mL water, or<br>• 33.5 g of sucrose in 300 mL water, or<br>• 55.8 mg of sucralose in 300 mL water, or<br>• 300 mL water,<br>subsequent *ad libitum* test meal.	Acute	Healthy participants;<br>BMI range 19.6 - 24.8 kg/m^2	Comparison between treatments:<br>• Subsequent energy intake: erythritol < sucrose, sucralose, or water preloads<br>• Total energy intake: erythritol < sucrose, sucralose, or water preloads
D-allulose				
	No human studies available.			

Table S4: Effects on glycemic control

Name	Route and Dose of Administration	Duration of Administration	Participants	Observed Effects
**Sucrose/glucose/fructose**				
Tai et al. 2010<br>*Appetite*	Oral administration of:<br>• 100 g sucrose in 300 mL water, or<br>• 300 mL water.	Acute	Healthy participants;<br>BMI mean: 22.4 ± 0.5 kg/m^2	Sucrose:<br>• ↑ Blood glucose; ↑ Insulin
Yunker et al. 2021,<br>*JAMA Network Open*	Oral administration of:<br>• 75 g sucrose in 300 mL water, or<br>• 178 - 358 mg sucralose (individually matched) in 300 mL water, or<br>• 300 mL water.	Acute	Healthy participants;<br>BMI range: 19.2 - 40.3 kg/m^2	Sucrose:<br>• ↑ Blood glucose; ↑ Insulin<br>Sucralose:<br>• No change in blood glucose and insulin
Ma et al. 2009,<br>*American Journal of Physiology Gastrointestinal and Liver Physiology*	Intragastric administration of:<br>• 50 g sucrose in 500 mL saline<br>• 80 mg sucralose in 500 mL saline, or<br>• 800 mg sucralose in 500 mL saline, or<br>• 500 mL saline.	Acute	Healthy participants;<br>BMI mean: 21.6 ± 1.2 kg/m^2	Sucrose:<br>• ↑ Blood glucose; ↑ Insulin<br>Sucralose:<br>• No change in blood glucose and insulin
Tey et al. 2017<br>*International Journal of Obesity*	Oral administration of preloads with:<br>• 65 g sucrose in 500 mL water, or<br>• 440 mg aspartame in 500 mL water, or<br>• 0.63 g monk fruit in 500 mL water, or<br>• 0.33 g stevia in 500 mL water,<br>subsequent *ad libitum* test meal.	Acute	Healthy participants;<br>BMI mean: 21.7 ± 1.9 kg/m^2	Sucrose:<br>• ↑ Blood glucose; ↑ Insulin after preload<br>Aspartame:<br>• No change in blood glucose and insulin after preload<br>Monk fruit:<br>• No change in blood glucose and insulin after preload<br>Stevia:<br>• No change in blood glucose and insulin after preload<br><br>Comparion between treatments:<br>• Increase in blood glucose and insulin overall (i.e. preload & test meal, 3h-AUC): sucrose = aspartame = monk fruit = stevia
Meyer-Gerspach et al. 2018	Intragastric administration of:<br>• 50 g glucose in 250 mL water, or<br>• 25 g fructose in 250 mL water, or	Acute	Healthy participants;<br>BMI range: 19 - 25 kg/m^2	Glucose:<br>• ↑ Blood glucose<br>Fructose:

American Journal of Clinical Nutrition	• 220 mg acesulfame K in 250 mL water, or<br>• 250 mL water.			• ↑ Blood glucose<br>Acesulfame K:<br>• No change in blood glucose<br><br>Comparison between treatments:<br>• Increase in blood glucose: glucose > fructose
Yau et al. 2017<br>*Nutrients*	Oral administration of:<br>• 36 g sucrose in 600 mL water, or<br>• 39.6 g glucose in 600 mL water, or<br>• 36 g fructose in 600 mL water, or<br>• 19.8 g glucose & 18 g fructose in 600 mL water.	Acute	Healthy participants;<br>BMI mean: 25.5 ± 3.8 kg/m^2	Sucrose:<br>• ↑ Blood glucose; ↑ Insulin<br>Glucose:<br>• ↑ Blood glucose; ↑ Insulin<br>Fructose:<br>• ↑ Blood glucose; ↑ Insulin<br>Glucose & Fructose:<br>• ↑ Blood glucose; ↑ Insulin<br><br>Comparison between treatments:<br>• Increase in blood glucose: sucrose, glucose, or glucose & fructose > fructose<br>• Increase in blood insulin: glucose > sucrose, or glucose & fructose > fructose
Kawakami et al. 2023<br>*Journal of Clinical Biochemistry and Nutrition*	Oral administration of:<br>• 50 g sucrose, or<br>• 25 g fructose, or<br>• 50 g isomaltulose<br>All drinks made up in water to a final volume of 250 mL.	Acute	Healthy participants;<br>BMI mean: 21.9 ± 3.0 kg/m^2	Sucrose:<br>• ↑ Blood glucose<br>Fructose:<br>• ↑ Blood glucose<br>Isomaltulose:<br>• ↑ Blood glucose<br><br>Comparison between treatments:<br>• Increase in blood glucose: sucrose, or isomaltulose > fructose
Jameel et al. 2014<br>*Lipids in Health and Disease*	Oral administration of:<br>• 50 g sucrose in 300 mL water, or<br>• 50 g glucose in 300 mL water, or<br>• 50 g fructose in 300 mL water.	Acute	Healthy participants;<br>BMI mean: 24.2 ± 0.7 kg/m^2	Sucrose:<br>• ↑ Blood glucose; ↑ Insulin<br>Glucose:<br>• ↑ Blood glucose; ↑ Insulin<br>Fructose:<br>• ↑ Blood glucose; ↑ Insulin

				Comparison between treatments:<br>• Increase in blood glucose and insulin: sucrose, or glucose > fructose
	•			•
Wölnerhanssen et al. 2015<br>*PLoS ONE*	Intragastric administration of:<br>• 75 g glucose in 300 mL water, or<br>• 25 g fructose in 300 mL water, or<br>• 300 mL water.	Acute	Healthy participants;<br>BMI range: 21.0 - 24.0 kg/m^2	Glucose:<br>• ↑ Blood glucose; ↑ Insulin<br>Fructose:<br>• No change in blood glucose and insulin
Kong et al. 1999<br>*Peptides*	Oral administration of:<br>• 75 g glucose in 300 mL water, or<br>• 75 g fructose in 300 mL water, or<br>• 75 g glucose followed by 75 g fructose 1h later.	Acute	Healthy participants;<br>BMI mean: 24.3 ± 2.9 kg/m^2	Glucose:<br>• ↑ Blood glucose; ↑ Insulin<br>Fructose:<br>• ↑ Blood glucose; ↑ Insulin<br><br>Comparison between treatments:<br>• Increase in blood glucose and insulin: glucose > fructose
Eckstein et al. 2021<br>*Nutrients*	Oral administration of:<br>• 76.3 g glucose in 300 mL water, or<br>• 76.3 g of fructose in 300 mL water, or<br>• 38.2 g of glucose & fructose in 300 mL water, or<br>• 0.2 g sucralose in 300 mL water	Acute	Healthy participants;<br>BMI mean: 23.7 ± 1.7 kg/m^2	Glucose:<br>• ↑ Blood glucose; ↑ Insulin<br>Fructose:<br>• ↑ Blood glucose; ↑ Insulin<br>Glucose & fructose:<br>• ↑ Blood glucose; ↑ Insulin<br>Sucralose:<br>• No change in blood glucose and insulin<br><br>Comparison between treatments:<br>• Increase in blood glucose and insulin:<br>glucose = glucose & fructose > fructose > sucralose

Yunker et al. 2021<br>*The Journal of Clinical Endocrinology & Metabolism*	Oral administration of:<br>• 75 g sucrose in 300 mL water, or<br>• 75 g glucose in 300 mL water.	Acute	Healthy participants;<br>BMI mean: 27.0 ± 5.0 kg/m^2	Sucrose:<br>• ↑ Blood glucose; ↑ Insulin<br>Glucose:<br>• ↑ Blood glucose; ↑ Insulin<br><br>Comparison between treatments:<br>• Increase in blood glucose and insulin: glucose > sucrose
Sigala et al. 2021<br>*The Journal of Clinical Endocrinology & Metabolism*	Daily oral administration (3x/day) of[1]:<br>• 147 g sucrose in 980 mL water (corresponding to 25% of daily energy requirement), or<br>• 150 g HFCS in 1000 mL water (corresponding to 25% of daily energy requirement), or<br>• 375 mg aspartame in ca. 1059 mL water,<br>OGTT at baseline and after 2 weeks.[1]	Chronic (2 weeks)	Healthy participants;<br>BMI range: 18 - 35 kg/m^2	Sucrose:<br>• ↓ Matsuda Index<br>• No change in fasting blood glucose, fasting insulin, HOMA-IR, and blood glucose during OGTT<br>• ↑ Insulin during OGTT<br>HFCS:<br>• ↓ Matsuda Index<br>• No change in fasting blood glucose, fasting insulin, and HOMA-IR<br>• ↑ Blood glucose; ↑ Insulin during OGTT<br>Aspartame:<br>• No change in Matsuda Index, fasting blood glucose, fasting insulin, HOMA-IR, and blood glucose and insulin during OGTT<br><br>Comparison between treatments:<br>• Increase in insulin during OGTT: sucrose = HFCS
Stanhope et al. 2009<br>*The Journal of Clinical Investigation*	Daily oral administration of:<br>• Glucose in water (corresponding to 25% of daily energy requirement), or<br>• Fructose in water (corresponding to 25% of daily energy requirement)	Chronic (10 weeks)	Participants with overweight and obesity;<br>BMI range: 25 - 35 kg/m^2	Glucose:<br>• ↓ Fasting blood glucose<br>• ↑ Fasting blood insulin<br>• ↑ Blood glucose, insulin, and insulin sensitivity index during OGTT

[1] Data provided by the corresponding author directly, not reported as such in the original publication.

	OGTT at baseline and after 10 weeks.			Fructose:<br>• ↑ Fasting blood glucose and insulin<br>• ↑ Blood glucose and insulin during OGTT<br>• ↓ Insulin sensitivity index during OGTT
Maersk et al. 2012<br>*American Journal of Clinical Nutrition*	Daily oral administration of:<br>• 106 g sucrose in 1000 mL water (regular cola), or<br>• 47 g carbohydrates in 1000 mL isocaloric semi-skimmed milk (carbohydrates 4.7 g/100 mL; protein 3.4 g/100 mL; fat 1.5 g/100 mL), or<br>• Aspartame 1000 mL water (diet cola), or<br>• 1000 mL water<br>OGTT at baseline and after 3 and 6 months.	Chronic (6 months)	Participants with overweight and obesity;<br>BMI range: 26 - 40 kg/m^2	Sucrose:<br>• No change in fasting blood glucose, insulin, and HOMA-IR<br>Semi-skimmed milk:<br>• No change in fasting blood glucose, insulin, and HOMA-IR<br>Aspartame diet cola:<br>• No change in fasting blood glucose, insulin, and HOMA-IR
Geidl et al. 2021<br>*Journal of Hepatology*	Daily oral administration (3x/day) of:<br>• 26.6 g sucrose in 200 mL water (total: 79.8 g/day)<br>• 26.6 g glucose in 200 mL water (total: 79.8 g/day)<br>• 26.6 g fructose in 200 mL water (total: 79.8 g/day)<br>• No beverage<br>OGTT at baseline and after 7 weeks	Chronic (7 weeks)	Healthy participants;<br>BMI mean: 21.8 ± 1.6 kg/m^2	Sucrose:<br>• No change in fasting blood glucose, fasting insulin, fasting HOMA-IR, and glucose or insulin during OGTT<br>Glucose:<br>• No change in fasting blood glucose, fasting insulin, fasting HOMA-IR, and glucose or insulin during OGTT<br>Fructose:<br>• No change in fasting blood glucose, fasting insulin, fasting HOMA-IR, and glucose or insulin during OGTT
Matikainen et a. 2017<br>*Nutrition, Metabolism & Cardiovascular Diseases*	Daily oral administration (3x/day) of:<br>• 25 g fructose in 330 mL water (total: 75 g /day)<br>Mixed meal test and OGTT at baseline and after 12 weeks.	Chronic (12 weeks)	Participants with overweight and obesity;<br>BMI range: 26.5-38.2 kg/m^2	Fructose:<br>• No change in fasting blood glucose, fasting insulin, fasting HOMA-IR, and Matusuda index during OGTT
Sucralose				

Ma et al. 2009, *American Journal of Physiology Gastrointestinal and Liver Physiology*	Intragastric administration of:<br>• 80 mg sucralose in 500 mL saline, or<br>• 800 mg sucralose in 500 mL saline, or<br>• 50 g sucrose in 500 mL saline, or<br>• 500 mL saline.	Acute	Healthy participants;<br>BMI mean: 21.6 ± 1.2 kg/m^2	Sucralose:<br>• No change in blood glucose and insulin<br>Sucrose:<br>• ↑Blood glucose; ↑Insulin
Yunker et al. 2021, *JAMA Network Open*	Oral administration of:<br>• 178 - 358 mg sucralose (individually matched) in 300 mL water, or<br>• 75 g sucrose in 300 mL water, or<br>• 300 mL water.	Acute	Healthy participants;<br>BMI range: 19.2 - 40.3 kg/m^2	Sucralose:<br>• No change in blood glucose and insulin<br>Sucrose:<br>• ↑Blood glucose; ↑Insulin
Steinert et al. 2011 *British Journal of Nutrition*	Intragastric administration of:<br>• 62 mg sucralose in 250 mL water, or<br>• 250 mL water.	Acute	Healthy participants;<br>BMI mean: 23.0 ± 0.5 kg/m^2	Sucralose:<br>• No change in blood glucose and insulin
Sylvetsky et al. 2016 *Nutrition & Metabolism*	Oral administration of preloads with:<br>• 68 mg sucralose in 355 mL water, or<br>• 170 mg sucralose in 355 mL water, or<br>• 250 mg sucralose in 355 mL water, or<br>• 355 mL water,<br>followed by an OGTT.	Acute	Healthy participants;<br>BMI mean: 25.8 ± 4.2 kg/m^2	Sucralose:<br>• No change in blood glucose, insulin, and c-peptide after preload<br><br>Comparison between treatments:<br>• Increase in blood glucose, insulin, and C-peptide during OGTT: sucralose (all concentrations) = water
Wu et al. 2013 *Diabetes Care*	Oral administration of preloads with:<br>• 52 mg sucralose in 240 mL water, or<br>• 200 mg acesulfame K in 240 mL water, or<br>• 46 mg sucralose & 26 mg acesulfame K in 240 mL water, or<br>• 240 mL water,<br>followed by an OGTT.	Acute	Healthy participants;<br>BMI mean: 25.5 ± 1.0 kg/m^2	Sucralose:<br>• No change in blood glucose and insulin after preload<br>Acesulfame K:<br>• No change in blood glucose and insulin after preload<br>Sucralose & acesulfame K:<br>• No change in blood glucose and insulin after preload<br><br>Comparison between treatments:<br>• Increase in blood glucose and insulin during OGTT: sucralose = acesulfame K = sucralose & acesulfame K

Wu et al. 2012<br>*American Journal of Clinical Nutrition*	Oral administration of preloads with:<br>• 60 mg sucralose in 400 mL water, or<br>• 40 g glucose in 400 mL water, or<br>• 40 g 3-OMG in 400 mL water, or<br>• 40 g tagatose & isomalt in 400 mL water,<br>followed by a labeled mashed potato meal containing ^{13}C octanoic acid.	Acute	Healthy participants;<br>BMI mean: 25.5 ± 1.5 kg/m^2	Sucralose:<br>• No change in blood glucose and insulin after preload<br>Glucose:<br>• ↑ Blood glucose; ↑ Insulin after preload<br>3-O-methyl-glucose:<br>• No change in blood glucose and insulin after preload<br>Tagatose & isomalt:<br>• No change in blood glucose and insulin after preload<br><br>Comparison between treatments:<br>• Increase in blood glucose during test meal (30 min): glucose > tagatose & isomalt, or sucralose > 3-OMG<br>• Increase in blood insulin during test meal (240 min): glucose > 3-OMG, or sucralose, or tagatose & isomalt
Ma et al. 2010<br>*British Journal of Nutrition*	Intraduodenal administration of:<br>• 960 mg sucralose in 600 mL saline, or<br>• 600 mL saline.	Acute	Healthy participants<br>BMI mean: 23.4 ± 0.8 kg/m^2	Sucralose<br>• No change in blood glucose
Ford et al. 2011<br>*European Journal of Clinical Nutrition*	Oral administration of:<br>• 4.15 mg sucralose in 50 mL water (i.e. 0.083% sucralose solution), or<br>• 4.15 mg sucralose & 25 g maltodextrin in 50 mL water (i.e. 0.083% sucralose solution & 50% maltodextrin solution), or<br>• 50 mL water.	Acute	Healthy participants;<br>BMI range: 18.8 - 23.9 kg/m^2	Sucralose:<br>• No change in blood glucose and insulin<br>Sucralose & maltodextrin:<br>• ↑ Blood glucose; ↑ Insulin
Brown et al. 2012<br>*Diabetes Care*	Oral administration of preloads with:<br>• 45.6 mg sucralose in 240 mL diet soda (mean concentration of sucralose: 190 ± 38 μg/mL), or<br>• 240 mL carbonated water,<br>followed by an OGTT.	Acute	Healthy participants;<br>BMI range: 19.1 - 35.9 kg/m^2	Comparison between treatments:<br>• Increase in blood glucose and C-peptide during OGTT: sucralose = water

Brown et al. 2009<br>*Diabetes Care*	Oral administration of preloads with:<br>• 45.6 mg sucralose in 240 mL diet soda (mean concentration of sucralose: 190 ± 38 µg/mL), or<br>• 240 mL carbonated water,<br>followed by an OGTT.	Acute	Healthy participants;<br>BMI mean: 25.6 ± 4.6 kg/m^2	Comparison between treatments:<br>• Increase in blood glucose, insulin, and C-peptides during OGTT: sucralose = water
Brown et al. 2011<br>*Nutrition Research*	Oral administration of:<br>• 6000 mg sucralose in 355 mL water, or<br>• 50 g sucrose in 355 mL water, or<br>• 50 g sucrose & 6 g sucralose in 355 mL water, or<br>• 355 mL water.	Acute	Healthy participants;<br>BMI mean: 22.1 ± 1.7 kg/m^2	Sucralose:<br>• No change in blood glucose and insulin<br>Sucrose:<br>• ↑ Blood glucose; ↑ Insulin<br>Sucrose & sucralose:<br>• No change in blood glucose<br>• ↑ Insulin<br><br>Comparison between treatments:<br>Increase in insulin: sucrose = sucrose & sucralose
Baird et al. 2000<br>*Food and Chemical Toxicology*<br>*(study 2)*	Daily oral administration (2x/d) of:<br>• 62.5 mg sucralose in water (total: 125 mg/day) during weeks 1–3,<br>• 125 mg sucralose in water (total: 250 mg/day) during weeks 4–7,<br>• 250 mg sucralose in water (total: 500 mg/day) during weeks 8–12.	Chronic (12 weeks)	Healthy participants;<br>Body weight mean: 71.5 kg	Sucralose:<br>• No change in fasting blood glucose and insulin
Baird et al. 2000<br>*Food and Chemical Toxicology*<br>*(study 1)*	Daily oral administration of ascending doses sucralose in 100mL water:<br>• 0 mg/kg on day 1, 1 mg/kg (ca. 70 mg) on day 3, 2.5 mg/kg (ca. 175 mg) on day 5, 5 mg/kg (ca. 385 mg) on day 7, and 10 mg/kg (ca. 700 mg) on day 9.<br>• No doses on day 0, 2, 4, 6, and 8.<br>• 2 mg/kg (ca. 140 mg) on day 11, 12, and 13.<br>• 5 mg/kg (ca. 350 mg) on day 14, 15, 16, and 17.	Chronic (17 days)	Healthy participants;<br>Body weight mean: 70 kg	Sucralose:<br>• No change in fasting blood glucose and insulin

Grotz et al. 2017 *Regulatory Toxicology and Pharmacology*	Daily oral administration (3x/day) of:<br>• 333.3 mg sucralose (total: 1 g/day) in capsules, or<br>• cellulose in capsules (placebo).	Chronic (12 weeks)	Healthy participants, BMI range: 19.4 - 27.0 kg/m^2	Sucralose:<br>• No change in fasting blood glucose, insulin, and HbA1C
Temizkan et al. 2015 *European Journal of Clinical Nutrition*	Oral administration of preloads with:<br>• 24 mg sucralose in 200 mL water, or<br>• 200 mL water,<br>followed by an OGTT.	Acute	Healthy participants; BMI mean: 30.3 ± 4.5 kg/m^2	Comparion between treatments:<br>• Increase in blood glucose during OGTT: sucralose < water<br>• Increase in insulin and C-peptide during OGTT: sucralose = water
Lertrit et al. 2018 *Nutrition*	Daily oral administration (1x/day) of:<br>• 200 mg sucralose in capsules, or<br>• empty capsules (placebo),<br>OGTT, or IVGTT at baseline and after 4 weeks.	Chronic (4 weeks)	Healthy participants; BMI range: 18.5 - 27.0 kg/m^2	Sucralose:<br>• ↓ Matsuda index<br>• ↑ HOMA-IR<br>Placebo:<br>• No change in Matsuda index and HOMA-IR<br><br>Comparison between treatments:<br>• Increase in blood glucose during OGTT and IVGTT: sucralose = placebo<br>• Increase in insulin during IVGTT (0-10 min): sucralose < placebo
Dalenberg et al. 2020 *Cell Metabolism*	Daily oral administration of:<br>• 60 mg sucralose in 355 mL beverage, or<br>• 30.38 g sucrose in 355 mL beverage, or<br>• 60 mg sucralose & 31.83 g maltodextrin in 355 mL beverage.<br>OGTT at baseline and after 2 weeks.	Chronic (10 days)	Healthy participants; BMI mean: 23.7 ± 3.1 kg/m^2	Sucralose:<br>• No change in insulin sensitivity<br>Sucrose:<br>• No change in insulin sensitivity<br>Sucralose & maltodextrin:<br>• ↓ Insulin sensitivity
Suez et al. 2022 *Cell*	Daily oral administration (3x/day) of:<br>• 204 mg sucralose (total: 612 mg/day) & 11.796 g glucose as a bulking agent (total: 35.388 g/day), or<br>• 11.796 g glucose alone (total: 35.388 g/day).<br>OGTT at baseline and after 2 weeks.	Chronic (2 weeks)	Healthy participants; BMI range: 18 - 28 kg/m^2	Sucralose & glucose:<br>• ↑ Blood glucose during OGTT<br>Glucose:<br>• ↑ Blood glucose during OGTT<br><br>Comparison between treatments:<br>• Increase in blood glucose during OGTT: sucralose & glucose > glucose
Xylitol				

Meyer-Gerspach et al. 2021 *Nutrients*	Intragastric administration of:<br>• 7 g xylitol in 300 mL water, or<br>• 17 g xylitol in 300 mL water, or<br>• 35 g xylitol in 300 mL water, or<br>• 300 mL water.	Acute	Healthy participants; BMI range 19.4 - 23.0 kg/m²	Xylitol:<br>• Dose-dependent ↑ Blood glucose; ↑ Insulin
Wölnerhanssen et al. 2016 *American Journal of Physiology Endocrinology and Metabolism*	Intragastric administration of:<br>• 50 g xylitol in 300 mL water, or<br>• 75 g glucose in 300 mL water, or<br>• 300 mL water.	Acute	Healthy participants and participants with obesity; BMI range 19.9 - 48.2 kg/m²	Xylitol:<br>• ↑ Blood glucose; ↑ Insulin<br>Glucose:<br>• ↑ Blood glucose; ↑ Insulin<br><br>Comparison between treatments:<br>• Increase in blood glucose and insulin: glucose > xylitol
Meyer-Gerspach et al. 2022 *Nutritional Neuroscience*	Intragastric administration of:<br>• 50 g xylitol in 300 mL water, or<br>• 75 g glucose in 300 mL water, or<br>• 300 mL water.	Acute	Healthy participants; BMI range: 20.0 - 38.9 kg/m²	Xylitol:<br>• ↑ Blood glucose; ↑ Insulin<br>Glucose:<br>• ↑ Blood glucose; ↑ Insulin<br><br>Comparison between treatments:<br>• Increase in blood glucose and insulin: glucose > xylitol
Müller-Hess et al. 1975 *Transfusion Medicine and Hemotherapy*	Oral administration of:<br>• 30 g xylitol in 300mL water, or<br>• 50 g xylitol in 300 mL water, or<br>• 50 g glucose in 300 mL water.	Acute	Healthy participants	Xylitol:<br>• ↑ Blood glucose; ↑ Insulin<br>Glucose:<br>• ↑ Blood glucose; ↑ Insulin<br><br>Comparison between treatments:<br>• Increase in blood glucose and insulin: glucose > xylitol
Natah et al. 1997 *American Journal of Clinical Nutrition*	Oral administration of:<br>• 25 g xylitol in 250 mL water, or<br>• 25 g glucose in 250 mL water.	Acute	Healthy participants; BMI mean: 22.1 ± 0.5 kg/m²	Xylitol:<br>• ↑ Blood glucose; ↑ Insulin<br>Glucose:<br>• ↑ Blood glucose; ↑ Insulin<br><br>Comparison between treatments:<br>• Increase in blood glucose and insulin: glucose > xylitol

Bordier et al. 2023 *BMJ Nutrition, Prevention & Health*	Daily oral administration (3x/d) of:<br>• 8 g xylitol in water (total: 24 g/day), or<br>• no substance (control).<br>OGTT at baseline and after 5 weeks.	Chronic (5 weeks)	Participants with obesity;<br>Xylitol group: BMI mean: 34.8 ± 2.8 kg/m^2<br>Control group: BMI mean: 34.9 ± 3.7 kg/m^2	Xylitol:<br>• ↑ Blood glucose and insulin during OGTT<br>• No change in HOMA Index<br>No substance (control):<br>• ↑ Blood glucose and insulin during OGTT<br>• No change in HOMA Index<br><br>Comparison between treatments:<br>• Increase in blood glucose and insulin during OGTT: xylitol = no substance
Erythritol				
Meyer-Gerspach et al. 2022 *Nutritional Neuroscience*	Intragastric administration of:<br>• 75 g erythritol in 300 mL water, or<br>• 75 g glucose in 300 mL water, or<br>• 300 mL water.	Acute	Healthy participants;<br>BMI range: 20.0 - 38.9 kg/m^2	Erythritol:<br>• No change in blood glucose and insulin<br>Glucose:<br>• ↑ Blood glucose; ↑ Insulin
Wölnerhanssen et al. 2016 *American Journal of Physiology Endocrinology and Metabolism*	Intragastric administration of:<br>• 75 g erythritol in 300 mL water, or<br>• 75 g glucose in 300 mL water, or<br>• 300 mL water.	Acute	Healthy participants and participants with obesity;<br>BMI range 19.9 - 48.2 kg/m^2	Erythritol:<br>• No change in blood glucose and insulin<br>Glucose:<br>• ↑ Blood glucose; ↑ Insulin<br><br>Comparison between treatments:<br>• Increase in blood glucose: glucose > erythritol
Wölnerhanssen et al. 2021 *Diabetes, Obesity and Metabolism*	Intragastric administration of:<br>• 10 g erythritol in 300 mL water, or<br>• 20 g erythritol in 300 mL water, or<br>• 50 g erythritol in 300 mL water, or<br>• 300 mL water.	Acute	Healthy participants;<br>BMI range: 19.4 - 24.0 kg/m^2	Erythritol:<br>• No change in blood glucose and insulin
Teysseire et al. 2022 *Nutrients*	Oral administration of preloads with:<br>• 50 g erythritol in 300 mL water, or<br>• 33.5 g sucrose in 300 mL water, or<br>• 55.8 mg sucralose in 300 mL water, or<br>• 300 mL water,<br>subsequent *ad libitum* test meal.	Acute	Healthy participants;<br>BMI range 19.6 - 24.8 kg/m^2	Erythritol:<br>• No change in blood glucose and insulin after preload<br>Sucrose:<br>• ↑ Blood glucose; ↑ Insulin after preload<br>Sucralose:<br>• No change in blood glucose and insulin after preloads

				Comparison between treatments:<br>• Increase in glucose and insulin during test meal: sucrose > erythritol, or sucrlaose
Teysseire et al. 2023<br>*Nutrients*	Intragastric administration of:<br>• 50 g erythritol in 300 mL water, or<br>• 25 g D-allulose in 300 mL water, or<br>• 300 mL water.	Acute	Healthy participants;<br>BMI range: 19.1 - 24.3 kg/m^2	Erythritol:<br>• No change in blood glucose and insulin<br>D-allulose:<br>• ↓ Blood glucose<br>• No change in blood insulin
Bornet et al. 1996<br>*Regulatory Toxicology and Pharmacology*	Oral administration of:<br>• 56 - 78 g erythritol in 250 mL water (corresponding to 1g/kg body weight).	Acute	Healthy participants;<br>BMI range 19.0 - 24.0 kg/m^2	Erythritol:<br>• No change in blood glucose and insulin
Bornet et al. 1996<br>*Regulatory Toxicology and Pharmacology*	Oral administration of a snack containing:<br>• 28 g erythritol (corresponding to 0.4 g/kg body weight of erythritol), or<br>• 56 g erythritol (corresponding to 0.8 g/kg body weight of erythritol), or<br>• 28 g sucrose (corresponding to 0.8 g/kg body weight of sucrose).	Acute	Healthy participants;<br>Group 0.4 g/kg BW: BMI mean: 22.5 ± 2.6 kg/m^2;<br>Group 0.8 g/kg BW: BMI mean: 22.0 ± 1.9 kg/m^2	Erythritol:<br>• No change in blood glucose and insulin<br>Sucrose:<br>• ↑ Insulin
Bordier et al. 2023<br>*BMJ Nutrition, Prevention & Health*	Daily oral administration (3x/day) of:<br>• 12 g erythritol in water (total: 36 g/day), or<br>• no substance (control),<br>OGTT at baseline and after 4 weeks.	Chronic (5 weeks)	Participants with obesity;<br>Erythritol group: BMI mean: 33.9 ± 3.7 kg/m^2,<br>Control group: BMI mean: 34.9 ± 3.7 kg/m^2	Erythritol:<br>• ↑ Blood glucose and insulin during OGTT<br>• No change in HOMA Index<br>No substance (control):<br>• ↑ Blood glucose and insulin during OGTT<br>• No change in HOMA Index<br><br>Comparison between treatments:<br>Increase in blood glucose and insulin during OGTT: erythritol = no substance
D-allulose				
Braunstein et al. 2018<br>*Nutrients*	Oral administration of:<br>• 5 or 10 g D-allulose & 75 g glucose in 500 mL water, or	Acute	Healthy participants;<br>BMI range: 18.5 - 30.0 kg/m^2	D-allulose & glucose:<br>• ↑ Blood glucose; ↑ Insulin<br>Fructose & glucose:<br>• ↑ Blood glucose; ↑ Insulin

	• 5 or 10 g fructose & 75 g glucose in 500 mL water, or<br>• 75 g glucose in 500 mL water.			Glucose:<br>• ↑ Blood glucose; ↑ Insulin<br><br>Comparison between treatments:<br>• Increase in blood glucose and insulin: D-allulose & glucose = fructose & glucose = glucose
Iida et al. 2008<br>*Journal of Nutritional Science and Vitaminology*	Oral administration of:<br>• 7.5 g D-allulose in 100 mL water, or<br>• 75 g maltodextrin in 300 mL water. or<br>• 75 g maltodextrin & 2.5 g D-allulose in 300 mL water, or<br>• 75 g maltodextrin & 5 g D-allulose in 300 mL water, or<br>• 75 g maltodextrin & 7.5 g D-allulose in 300 mL water.	Acute	Healthy participants;<br>BMI mean: 20.7 ± 1.8 kg/m^2	D-allulose:<br>• No change in blood glucose and insulin<br>Maltodextrin:<br>• ↑ Blood glucose; ↑ Insulin<br>D-allulose & maltodextrin:<br>• ↑ Blood glucose; ↑ Insulin<br><br>Comparion between treatments :<br>• Increase in blood glucose and insulin: D-allulose (≥ 5 g) & maltodextrin < maltodextrin
Hayashi et al. 2010<br>*Bioscience, Biotechnology, and Biochemistry*	Daily oral administration (3x/day) of:<br>• 5 g D-allulose in 200 mL tea consumed with meals (total: 15 g/day), or<br>• 5 g glucose in 200 mL tea consumed with meals (total: 15 g/day).	Chronic (12 weeks)	Healthy participants;<br>D-allulose group: BMI mean: 21.3 ± 2.2 kg/m·;<br>Glucose group: BMI mean: 21.5 ± 3.0 kg/m^2	D-allulose:<br>• No change in blood glucose and insulin during meal<br>Glucose:<br>• No change in blood glucose and insulin during meal
Teysseire et al. 2023<br>*Nutrients*	Intragastric administration of:<br>• 25 g D-allulose in 300 mL water, or<br>• 50 g erythritol in 300 mL water, or<br>• 300 mL water.	Acute	Healthy participants;<br>BMI range: 19.1 - 24.3 kg/m2	D-allulose:<br>• ↓ Blood glucose<br>• No change in blood insulin<br>Erythritol:<br>• No change in blood glucose and insulin
Kimura et al. 2017<br>*Nutrition*	Oral administration of:<br>• 5 g D-allulose in 150 mL water, or<br>• 10 mg aspartame in 150 mL water .<br>followed by a test meal.	Acute	Healthy participants;<br>BMI mean: 20.9 ± 0.7 kg/m^2	Comparison between treatments:<br>• Increase in blood glucose during test meal: D-allulose < aspartame<br>• Increase in insulin during test meal: D-allulose = aspartame
Franchi et al. 2021<br>*BMJ Open Diabetes Research & Care*	Oral administration of:<br>• 2.5 g D-allulose & 50 g sucrose in 300 mL water, or	Acute	Healthy participants;<br>BMI mean: 28.0 ± 4.0 kg/m^2	D-allulose & sucrose:<br>• ↑ Blood glucose; ↑ Insulin<br>Sucrose:<br>• ↑ Blood glucose; ↑ Insulin

- 5 g D-allulose & 50 g sucrose in 300 mL water, or
- 7.5 g D-allulose & 50 g sucrose in 300 mL water, or
- 10 g D-allulose & 50 g sucrose in 300 mL water, or
- 50 g sucrose in 300 mL water.

Comparison between treatments:

- Increase in blood glucose and insulin: D-allulose & sucrose < sucrose

Table S5: Effects on blood lipids

Name	Route and Dose of Administration	Duration of Administration	Participants	Observed Effects
**Sucrose/glucose/fructose**				
Jameel et al. 2014<br>*Lipids in Health and Disease*	Oral administration of:<br>• 50 g sucrose in 300 mL water, or<br>• 50 g glucose in 300 mL water, or<br>• 50 g fructose in 300 mL water.	Acute	Healthy participants;<br>BMI mean: 24.2 ± 0.7 kg/m²	Comparison between treatments:<br>• Increase in total cholesterol, LDL, and HDL: fructose > sucrose or glucose<br>• Increase in TG: sucrose = glucose = fructose
Yau et al. 2017<br>*Nutrients*	Oral administration of:<br>• 39.6 g of glucose in 600 mL water<br>• 36 g of fructose in 600 mL water, or<br>• with and without prior fructose supplementation (3 days with 120 g/d of fructose).	Acute	Healthy participants;<br>BMI mean: 25.5 ± 3.8 kg/m²	Glucose:<br>• No change in TG<br>Fructose:<br>• No change in TG
Chong et al. 2007<br>*American Journal of Clinical Nutrition*	Oral administration of test meals with:<br>• 52.5 g glucose (corresponding to 0.75 g/kg body weight glucose, or<br>• 52.5 g fructose (corresponding to 0.75 g/kg body weight fructose).	Acute	Healthy participants;<br>BMI range: 22 - 31 kg/m²	Glucose:<br>• ↑ TG<br>Fructose:<br>• ↑ TG<br><br>Comparison between treatments:<br>• Increase in TG: fructose > glucose
Bantle et al. 2000<br>*American Journal of Clinical Nutrition*	Daily oral administration of:<br>• 81 g/d glucose (corresponding to 17% of daily energy requirement), or<br>• 85 g/d fructose (corresponding to 17% of daily energy requirement).	Chronic (6 weeks)	Healthy participants;<br>BMI mean: between 24.6 and 25.8 depending on age and gender	Comparison between treatments:<br>• Fasting total cholesterol, LDL, and HDL: glucose = fructose<br>• Fasting, post prandial, and daylong TG: fructose > glucose (only in men)
Lê et al. 2006<br>*American Journal of Clinical Nutrition*	Daily oral administration of:<br>• 103 g/d fructose (corresponding to 1.5 g/kg body weight/d).	Chronic (4 weeks)	Healthy participants;<br>Mean body weight: 69.3 ± 2.6	Fructose:<br>• ↑ Fasting TG
Sigala et al. 2021<br>*The Journal of Clinical Endocrinology & Metabolism*	Daily oral administration of:<br>• 147 g sucrose in 980 mL water (corresponding to 25% of daily energy requirement), or	Chronic (2 weeks)	Healthy participants;<br>BMI range: 18 - 35 kg/m²	Sucrose:<br>• ↑ Fasting LDL; ↑ Fasting TG<br>HFCS:<br>• ↑ Fasting LDL; ↑ Fasting TG<br>Aspartame:<br>• No change in fasting LDL and TG

	• 150 g HFCS in 1000 mL water (corresponding to 25% of daily energy requirement), or<br>• 375 mg aspartame in ca. 1059 mL water,<br>OGTT at baseline and after 2 weeks.[2]			Comparison between treatments:<br>• Increase in fasting LDL and TG: sucrose = HFCS
Stanhope et al. 2009<br>*The Journal of Clinical Investigation*	Daily oral administration of:<br>• Glucose in water (corresponding to 25% of daily energy requirement), or<br>• Fructose in water (corresponding to 25% of daily energy requirement)<br>OGTT at baseline and after 10 weeks.	Chronic (10 weeks)	Participants with overweight and obesity;<br>BMI range: 25 - 35 kg/m^2	Glucose:<br>• No change in fasting total cholesterol and fasting LDL<br>• ↓ Fasting HDL; ↑ Fasting LDL<br>• ↑ Fasting TG; no change in 23h-AUC for TG<br>Fructose:<br>• ↑ Fasting total cholesterol; ↑ Fasting LDL; ↑ Fasting HDL; ↑ Fasting sLDL<br>• No change in fasting TG; ↑ 23h-AUC for TG
Maersk et al. 2012<br>*American Journal of Clinical Nutrition*	Daily oral administration of:<br>• 106 g sucrose in 1000 mL water (regular cola), or<br>• 47 g carbohydrates in 1000 mL isocaloric semi-skimmed milk (carbohydrates 4.7 g/100 mL; protein 3.4 g/100 mL; fat 1.5 g/100 mL), or<br>• Aspartame in 1000 mL water (diet cola), or<br>• 1000 mL water<br>OGTT at baseline and after 3 and 6 months.	Chronic (6 months)	Participants with overweight and obesity;<br>BMI range: 26 - 40 kg/m^2	Sucrose:<br>• ↑ Fasting total cholesterol; ↑ Fasting TG<br>• No change in fasting HDL<br>Semi-skimmed milk:<br>• No change in fasting total cholesterol, HDL and TG<br>Aspartame diet cola:<br>• No change in fasting total cholesterol, HDL and TG
Geidl et al. 2021<br>*Journal of Hepatology*	Daily oral administration (3x/day) of:<br>• 26.6 g sucrose in 200 mL water (total: 79.8 g/day)<br>• 26.6 g glucose in 200 mL water (total: 79.8 g/day)<br>• 26.6 g fructose in 200 mL water (total: 79.8 g/day)<br>• No beverage	Chronic (7weeks)	Healthy participants;<br>BMI mean: 21.8 ± 1.6 kg/m^2	Sucrose:<br>• No change in fasting total cholesterol, LDL, HDL, and TG; ↑ Fasting sdLDL<br>Glukose:<br>• No change in fasting total cholesterol, LDL, HDL, and TG<br>Fructose:

[2] Data provided by the corresponding author directly, not reported as such in the original publication

	OGTT at baseline and after 7 weeks			• No change in fasting total cholesterol, LDL, HDL, and TG
Matikainen et al. 2017<br>*Nutrition, Metabolism & Cardiovascular Diseases*	Daily oral administration (3x/day) of:<br>• 25 g fructose in 330 mL water (total: 75 g /day)<br>Mixed meal test and OGTT at baseline and after 12 weeks.	Chronic (12 weeks)	Participants with overweight and obesity;<br>BMI range: 26.5-38.2 kg/m^2	Fructose:<br>• ↑ Fasting TG and during the mixed meal
Sucralose				
Baird et al. 2000<br>*Food and Chemical Toxicology*<br>*(study 1)*	Daily oral administration of ascending doses sucralose in 100 mL water:<br>• 0 mg/kg on day 1, 1 mg/kg (ca. 70 mg) on day 3, 2.5 mg/kg (ca. 175 mg) on day 5, 5 mg/kg (ca. 385 mg) on day 7, and 10 mg/kg (ca. 700 mg) on day 9.<br>• No doses on day 0, 2, 4, 6, and 8.<br>• 2 mg/kg (ca. 140 mg) on day 11, 12, and 13.<br>• 5 mg/kg (ca. 350 mg) on day 14, 15, 16, and 17..	Chronic (17 days)	Healthy participants;<br>Body weight mean: 70 kg	Sucralose:<br>• No change in fasting total cholesterol and TG
Xylitol				
Meyer-Gerspach et al. 2021<br>*Nutrients*	Intragastric administration of:<br>• 7 g xylitol in 300 mL water, or<br>• 17 g xylitol in 300 mL water, or<br>• 35 g xylitol in 300 mL water, or<br>• 300 mL water.	Acute	Healthy participants<br>BMI range: 19.4 - 23.0 kg/m^2	Xylitol:<br>• No change in total cholesterol, LDL, HDL, and TG.
Bordier et al. 2023<br>*BMJ Nutrition, Prevention & Health*	Daily oral administration (3x/d) of:<br>• 8 g xylitol in water (total: 24 g/day), or<br>• no substance (control).	Chronic (5 weeks)	Participants with obesity;<br>Xylitol group: BMI mean: 34.8 ± 2.8 kg/m^2<br>Control group: BMI mean: 34.9 ± 3.7 kg/m^2	Xylitol :<br>• No change in fasting total cholesterol, HDL, and TG<br>• ↓ Fasting LDL<br>No substance (control):<br>• No change in fasting total cholesterol, HDL, and TG

				• ↓ Fasting LDL<br><br>Comparison between treatments :<br>• Decrease in fasting LDL: xylitol = no substance (control)
Erythritol				
Wölnerhanssen et al. 2021<br>*Diabetes, Obesity and Metabolism*	Intragastric administration of:<br>• 10 g erythritol in 300 mL water, or<br>• 20 g erythritol in 300 mL water, or<br>• 50 g erythritol in 300 mL water<br>• 300 mL water.	Acute	Healthy participants;<br>BMI range: 19.4 - 24.0 kg/m²	Erythritol:<br>• No change in total cholesterol, LDL, HDL, and TG
Teysseire et al. 2023<br>*Nutrients*	Intragastric administration of:<br>• 50 g erythritol in 300 mL water, or<br>• 25 g D-allulose in 300 mL water, or<br>• 300 mL water.	Acute	Healthy participants;<br>BMI range: 19.1 – 24.3 kg/m²	Erythritol:<br>• No change in total cholesterol, LDL, HDL, and TG<br>D-allulose:<br>• No change in total cholesterol, LDL, HDL, and TG
Bordier et al. 2023<br>*BMJ Nutrition, Prevention & Health*	Daily oral administration (3x/day) of:<br>• 12 g erythritol (total: 36 g/day) in water, or<br>• no substance (control).	Chronic (5 weeks)	Participants with obesity;<br>Erythritol group: BMI mean: 33.9 ± 3.7 kg/m²,<br>Control group: BMI mean: 34.9 ± 3.7 kg/m²	Erythritol:<br>• No change in fasting total cholesterol, HDL, and TG<br>• ↓ Fasting LDL<br>No substance (control):<br>• No change in fasting total cholesterol, HDL, and TG<br>• ↓ Fasting LDL<br><br>Comparison between treatments:<br>• Decrease in fasting LDL: erythritol = no substance (control)
D-Allulose				
Teysseire et al. 2023<br>*Nutrients*	Intragastric administration of:<br>• 25 g D-allulose in 300 mL water, or<br>• 50 g erythritol in 300 mL water, or<br>• 300 mL water	Acute	Healthy participants;<br>BMI range: 19.1 - 24.3 kg/m²	D-allulose:<br>• No change in total cholesterol, LDL, HDL, and TG<br>Erythritol:<br>• No change in total cholesterol, LDL, HDL, and TG

Kimura et al. 2017<br>*Nutrition*	Oral administration of preloads with:<br>• 5 g D-allulose in 150 mL water, or<br>• 10 mg aspartame in 150 mL water,<br>followed by a test meal.	Acute	Healthy participants;<br>BMI mean: 20.9 ± 0.7 kg/m^2	D-allulose:<br>• No change in total cholesterol, and TG during test meal<br>Aspartame:<br>• No change in total cholesterol, and TG during test meal
Hayashi et al. 2010<br>*Bioscience, Biotechnology, and Biochemistry*	Daily oral administration (3x/day) of:<br>• 5 g D-allulose in 200 mL tea consumed with meals (total: 15 g/day), or<br>• 5 g glucose in 200 mL tea consumed with meals (total: 15 g/day).	Chronic (12 weeks)	Healthy participants;<br>D-allulose group: BMI mean: 21.3 ± 2.2 kg/m^2;<br>Glucose group: BMI mean: 21.5 ± 3.0 kg/m^2	D-allulose:<br>• No change in fasting total cholesterol, LDL, and TG<br>Glucose:<br>• No change in fasting total cholesterol, LDL, and TG

Table S6: Effects on uric acid

Name	Route and Dose of Administration	Duration of Administration	Participants	Observed Effects
Glucose/Fructose/Sucrose				
Wolyniec et al. 2022<br>*Frontiers in Physiology*	Oral administration of:<br>• 35 g sucrose in 500 mL water, or<br>• 35 g glucose in 500 mL water, or<br>• 35 g fructose in 500 mL water, or<br>• 35 g xylitol in 500 mL water.	Acute	Healthy participants;<br>BMI mean: 25.7 ± 2.7 kg/m^2<br>(football players)	Sucrose:<br>• ↑ Blood uric acid<br>Glucose:<br>• ↑ Blood uric acid<br>Fructose:<br>• ↑ Blood uric acid<br>Xylitol:<br>• ↑ Blood uric acid<br><br>Comparison between treatments:<br>• Increase in blood uric acid: xylitol > sucrose, glucose, or fructose
Kawakami et al. 2023<br>*Journal of Clinical Biochemistry and Nutrition*	Oral administration of:<br>• 50 g sucrose, or<br>• 25 g fructose, or<br>• 50 g isomaltulose,<br>all drinks made up in water to a final volume of 250 mL.	Acute	Healthy participants;<br>BMI mean: 21.9 ± 3.0 kg/m^2	Sucrose:<br>• ↑ Blood uric acid<br>Fructose:<br>• ↑ Blood uric acid<br>Isomaltulose:<br>• ↑ Blood uric acid<br><br>Comparison between treatments:<br>• Increase in blood uric acid: fructose > sucrose or isomaltulose.
Sigala et al. 2021<br>*The Journal of Clinical Endocrinology & Metabolism*	Daily oral administration of:<br>• 147 g sucrose in 980 mL water (corresponding to 25% of daily energy requirement), or<br>• 150 g HFCS in 1000 mL water (corresponding to 25% of daily energy requirement), or<br>• 375 mg aspartame in ca. 1059 mL water,	Chronic (2 weeks)	Healthy participants;<br>BMI range: 18 - 35 kg/m^2	Sucrose:<br>• ↑ Blood uric acid (AUC over 24h)<br>HFCS:<br>• ↑ Blood uric acid (AUC over 24h)<br>Aspartame:<br>• No change in blood uric acid (AUC over 24h)<br><br>Comparison between treatments:

	OGTT at baseline and after 2 weeks.[3]			• Increase in blood uric acid: sucrose = HFCS
Bruun et al. 2015<br>*European Journal of Clinical Nutrition*	Daily oral administration of:<br>• 106 g sucrose in 1000 mL water (regular cola), or<br>• 47 g carbohydrates in 1000 mL isocaloric semi-skimmed milk (carbohydrates 4.7 g/100 mL; protein 3.4 g/100 mL; fat 1.5 g/100 mL), or<br>• Aspartame in 1000 mL water (diet cola), or<br>• 1000 mL water.	Chronic (6 months)	Participants with overweight and obesity; BMI mean: 32.1 ± 0.5 kg/m²	Sucrose:<br>• ↑ Fasting blood uric acid<br>Semi-skimmed milk:<br>• No change in fasting blood uric acid<br>Aspartame diet cola:<br>• No change in fasting blood uric acid
Cox et al. 2012<br>*Nutrition & Metabolism*	Daily oral administration of :<br>• glucose (corresponding to 25% of daily energy requirement), or<br>• fructose corresponding to 25% of daily energy requirement).	Chronic (10 weeks)	Participants with overweight and obesity; BMI range: 28.4 - 30.3 kg/m²	Glucose:<br>• ↑ Fasting blood uric acid<br>Fructose:<br>• ↑ Fasting blood uric acid<br><br>Comparison between treatments:<br>• Increase in fasting blood uric acid: fructose > glucose
Sucralose				
Baird et al. 2000<br>*Food and Chemical Toxicology (study 2)*	Daily oral administration (2x/d) of:<br>• 62.5 mg sucralose in water (total: 125 mg/day) during weeks 1–3,<br>• 125 mg sucralose in water (total: 250 mg/day) during weeks 4–7,<br>250 mg sucralose in water (total: 500 mg/day) during weeks 8–12.	Chronic (12 weeks)	Healthy participants<br>Body weight mean: 71.5 kg	Sucralose:<br>• No change in fasting blood uric acid
Xylitol				
Meyer-Gerspach et al. 2021<br>*Nutrients*	Intragastric administration of:<br>• 7 g xylitol in 300 mL water, or<br>• 17 g xylitol in 300 mL water, or	Acute	Healthy participants;<br>BMI range 19.4–23.0 kg/m²	Xylitol:<br>• ↑ Blood uric acid after 35 g xylitol

[3] Data provided by the corresponding author directly, not reported as such in the original publication

	• 35 g xylitol in 300 mL water, or<br>• 300 mL water.			
Wolyniec et al. 2022<br>*Frontiers in Physiology*	Oral administration of:<br>• 35 g xylitol in 500 mL water, or<br>• 35 g sucrose in 500 mL water, or<br>• 35 g glucose in 500 mL water, or<br>• 35 g fructose in 500 mL water.	Acute	Healthy participants;<br>BMI mean: 25.7 ± 2.7 kg/m^2<br>(football players)	Xylitol:<br>• ↑ Blood uric acid<br>Sucrose:<br>• ↑ Blood uric acid<br>Glucose:<br>• ↑ Blood uric acid<br>Fructose:<br>• ↑ Blood uric acid<br><br>Comparison between treatments:<br>• Increase in blood uric acid: xylitol > sucrose, glucose, or fructose
Bordier et al. 2023<br>*BMJ Nutrition, Prevention & Health*	Daily oral administration (3x/d) of:<br>• 8 g xylitol in water (total: 24 g/day), or<br>• no substance (control).	Chronic (5 weeks)	Participants with obesity;<br>Xylitol group: BMI mean: 34.8 ± 2.8 kg/m^2<br>Control group: BMI mean: 34.9 ± 3.7 kg/m^2	Xylitol:<br>• No change in fasting blood uric acid
Erythritol				
Wölnerhanssen et al. 2021<br>*Diabetes, Obesity and Metabolism*	Intragastric administration of:<br>• 10 g erythritol in 300 mL water, or<br>• 20 g erythritol in 300 mL water, or<br>• 50 g erythritol in 300 mL water, or<br>• 300 mL water..	Acute	Healthy participants;<br>BMI range: 19.4 - 24.0 kg/m^2	Erythritol:<br>• No change in blood uric acid
Teysseire et al. 2023<br>*Nutrients*	Intragastric administration of:<br>• 50 g erythritol in 300 mL water, or<br>• 25 g D-allulose in 300 mL water, or<br>• 300 mL water.	Acute	Healthy participants;<br>BMI range: 19.1 - 24.3 kg/m^2	Erythritol:<br>• No change in blood uric acid<br>D-allulose:<br>• No change in blood uric acid
Bordier et al. 2023<br>*BMJ Nutrition, Prevention & Health*	Daily oral administration (3x/d) of:<br>• 12 g erythritol in water (total: 36 g/day), or<br>• no substance (control).	Chronic (5 weeks)	Participants with obesity;<br>Erythritol group: BMI mean: 33.9 ± 3.7 kg/m^2,<br>Control group: BMI mean: 34.9 ± 3.7 kg/m^2	Erythritol:<br>• No change in fasting blood uric acid
D-allulose				

Teysseire et al. 2023<br>*Nutrients*	Intragastric administration of:<br>• 25 g D-allulose in 300 mL water, or<br>• 50 g erythritol in 300 mL water, or<br>• 300 mL water.	Acute	Healthy participants;<br>BMI range: 19.1 -2 4.3 kg/m^2	D-allulose:<br>• No change in blood uric acid<br>Erythritol:<br>• No change in blood uric acid
Hayashi et al. 2010<br>*Bioscience, Biotechnology, and Biochemistry*	Daily oral administration (3x/day) of:<br>• 5 g D-allulose in 200 mL tea consumed with meals (total: 15 g/day), or<br>• 5 g glucose in 200 mL tea consumed with meals (total: 15 g/day).	Chronic (12 weeks)	Healthy participants;<br>D-allulose group: BMI mean: 21.3 ± 2.2 kg/m^2;<br>Glucose group: BMI mean: 21.5 ± 3.0 kg/m^2	D-allulose:<br>• No change in fasting blood uric acid<br>Glucose:<br>• No change in fasting blood uric acid
